# Cultivation and Genomic Analysis of “*Candidatus* Nitrosocaldus islandicus,” an Obligately Thermophilic, Ammonia-Oxidizing Thaumarchaeon from a Hot Spring Biofilm in Graendalur Valley, Iceland

**DOI:** 10.3389/fmicb.2018.00193

**Published:** 2018-02-14

**Authors:** Anne Daebeler, Craig W. Herbold, Julia Vierheilig, Christopher J. Sedlacek, Petra Pjevac, Mads Albertsen, Rasmus H. Kirkegaard, José R. de la Torre, Holger Daims, Michael Wagner

**Affiliations:** ^1^Division of Microbial Ecology, Department of Microbiology and Ecosystem Science, Research Network “Chemistry meets Microbiology”, University of Vienna, Vienna, Austria; ^2^Department of Chemistry and Bioscience, Center for Microbial Communities, Aalborg University, Aalborg, Denmark; ^3^Department of Biology, San Francisco State University, San Francisco, CA, United States

**Keywords:** AOA, thaumarchaeota, thermophile, nitrification, hot spring, *nirK*, polymerase, anaerobic metabolism

## Abstract

Ammonia-oxidizing archaea (AOA) within the phylum Thaumarchaeota are the only known aerobic ammonia oxidizers in geothermal environments. Although molecular data indicate the presence of phylogenetically diverse AOA from the *Nitrosocaldus* clade, group 1.1b and group 1.1a Thaumarchaeota in terrestrial high-temperature habitats, only one^§^ enrichment culture of an AOA thriving above 50°C has been reported and functionally analyzed. In this study, we physiologically and genomically characterized a newly discovered thaumarchaeon from the deep-branching Nitrosocaldaceae family of which we have obtained a high (∼85%) enrichment from biofilm of an Icelandic hot spring (73°C). This AOA, which we provisionally refer to as “*Candidatus* Nitrosocaldus islandicus,” is an obligately thermophilic, aerobic chemolithoautotrophic ammonia oxidizer, which stoichiometrically converts ammonia to nitrite at temperatures between 50 and 70°C. “*Ca.* N. islandicus” encodes the expected repertoire of enzymes proposed to be required for archaeal ammonia oxidation, but unexpectedly lacks a *nirK* gene and also possesses no identifiable other enzyme for nitric oxide (NO) generation^§^. Nevertheless, ammonia oxidation by this AOA appears to be NO-dependent as “*Ca.* N. islandicus” is, like all other tested AOA, inhibited by the addition of an NO scavenger. Furthermore, comparative genomics revealed that “*Ca.* N. islandicus” has the potential for aromatic amino acid fermentation as its genome encodes an indolepyruvate oxidoreductase (*iorAB*) as well as a type 3b hydrogenase, which are not present in any other sequenced AOA. A further surprising genomic feature of this thermophilic ammonia oxidizer is the absence of DNA polymerase D genes^§^ – one of the predominant replicative DNA polymerases in all other ammonia-oxidizing Thaumarchaeota. Collectively, our findings suggest that metabolic versatility and DNA replication might differ substantially between obligately thermophilic and other AOA.

## Introduction

Thaumarchaeota (Brochier-Armanet et al., 2008) are among the most abundant archaeal organisms on Earth, and thrive in most oxic environments (Francis et al., 2007; Erguder et al., 2009; Schleper and Nicol, 2010; Bouskill et al., 2012; Prosser and Nicol, 2012; Stahl and de la Torre, 2012; Stieglmeier et al., 2014a), but have also been detected in anoxic systems (Molina et al., 2010; Bouskill et al., 2012; Buckles et al., 2013; Beam et al., 2014; Lin et al., 2015). This phylum comprises ammonia-oxidizing archaea (AOA) and other archaeal taxa in which ammonia oxidation has not been demonstrated. All cultured members of the Thaumarchaeota are AOA and grow by using ammonia, urea or cyanate as substrate (Palatinszky et al., 2015; Bayer et al., 2016; Sauder et al., 2017; Qin et al., 2017a), although *in situ* experiments suggest that certain members of this phylum capable of ammonia oxidation also possess other lifestyles (Mussmann et al., 2011; Sauder et al., 2017). In aquatic and terrestrial environments Thaumarchaeota often co-occur with ammonia-oxidizing bacteria (AOB), and frequently outnumber them by orders of magnitude (Francis et al., 2005; Leininger et al., 2006; Mincer et al., 2007; Adair and Schwartz, 2008; Abell et al., 2010; Mussmann et al., 2011; Zeglin et al., 2011; Daebeler et al., 2012). Thaumarchaeota also inhabit extreme environments like terrestrial hot springs and other high temperature habitats, where AOB are not detectable (Weidler et al., 2007; Reigstad et al., 2008; Wang et al., 2009; Zhao et al., 2011; Chen et al., 2016). In addition to the presence of Thaumarchaeota in hot environments, high *in situ* nitrification rates (Reigstad et al., 2008; Dodsworth et al., 2011; Chen et al., 2016) and transcription of genes involved in archaeal ammonia oxidation in several hot springs over 74°C (Zhang et al., 2008; Jiang et al., 2010) support an important role of thermophilic AOA in these systems.

Despite their apparent importance for nitrogen cycling in a wide range of thermal habitats, only one^§^ thermophilic AOA species from an enrichment culture has been reported to date (de la Torre et al., 2008; Qin et al., 2017b) and was named “*Candidatus* (*Ca*.) Nitrosocaldus yellowstonensis.” The authors note that throughout this study the definition for thermophiles by Stetter (1998) as organisms that grow optimally above 50°C is used. In addition, several enrichment cultures and one pure culture of moderately thermophilic AOA, which are able to grow at 50°C, but have growth optima only at temperatures below 50°C, have been described (Hatzenpichler et al., 2008; Lebedeva et al., 2013; Palatinszky et al., 2015). Therefore, our current knowledge on specific adaptations or metabolic capabilities of thermophilic AOA growing preferably at temperatures above 50°C is very limited (Spang et al., 2012).

In 16S rRNA and ammonia monooxygenase subunit A (*amoA*) gene trees “*Ca.* Nitrosocaldus yellowstonensis” branches most deeply among Thaumarchaeota that possess ammonia monooxygenase (AMO) genes. In consequence, the Nitrosocaldales clade has been considered as being close to the evolutionary origin of Thaumarchaeota encoding the genetic repertoire for ammonia oxidation (de la Torre et al., 2008; Spang et al., 2017). However, since the genome sequence of “*Ca.* N. yellowstonensis” is not yet published, phylogenomic analysis to confirm an ancestral position of the Nitrosocaldales relative to other Thaumarchaeota have been pending.

Here we report on the enrichment, phylogenomic analyses, and selected (putative) metabolic features of an obligately thermophilic AOA from the Nitrosocaldales clade obtained from a biofilm collected from a 73°C hot spring in Iceland. This organism, provisionally referred to as “*Ca.* Nitrosocaldus islandicus,” occupies a fundamentally different niche compared to other genomically characterized AOA as its ammonia-oxidizing activity is restricted to temperatures ranging from 50 to 70°C.

## Materials and Methods

### Enrichment, Cultivation, and Physiological Experiments

The enrichment of “*Ca.* N. islandicus” was initiated by inoculation of 40 ml sterile mineral medium (Koch et al., 2015) containing 0.5 mM filter-sterilized NH_4_Cl with approximately 0.1 g of hot spring biofilm, which had been submerged in running water at the sampling site in a geothermal area in Graendalur valley, (64° 1′ 7″ N, 21° 11′ 20″ W) Iceland. At the sampling site, the spring had a pH of 6.5 and a temperature of 73°C. The culture was initially incubated without agitation in 100 ml glass bottles in the dark at 60°C and checked weekly for ammonium and nitrite content of the medium by using Nessler’s reagent (K_2_HgI_4_ – KOH solution; Sigma–Aldrich) and nitrite/nitrate test stripes (Merckoquant; Merck). Ammonium (1 mM NH_4_Cl) was replenished when completely consumed. At the same time pH was monitored by using pH test stripes (Macherey-Nagel) and kept at pH 7–8 by titration with NaHCO_3_. When the pH dropped below 6 the enrichment culture ceased to oxidize ammonia, but activity was restored by readjusting the pH to between 7 and 8. The ammonium and nitrite concentrations were quantified photometrically (Kandeler and Gerber, 1988; Miranda et al., 2001) using an Infinite 200 Pro spectrophotometer (Tecan Group AG). The microbial community composition of the enrichment was regularly monitored by fluorescence *in situ* hybridization (FISH) with 16S rRNA-targeted probes labeled with dyes Cy3, Cy5, or Fluos as described elsewhere (Daims et al., 2005). Probes targeting most bacteria (EUB338 probe mix; Amann et al., 1990; Daims et al., 1999), most archaea (Arch915, Stahl and Amann, 1991) and most Thaumarchaeota (Thaum726, Beam, 2015) were applied. All positive results were verified using the nonsense probe non-EUB338 (Wallner et al., 1993) labeled with the same dyes. Additions of the antibiotics rifampicine, gentamicine, and kanamycine at concentrations ranging from 10 to 100 mg l^-1^ were applied alone and in combination, but no ammonia oxidizing activity was observed in the course of 8 weeks. Treatments with the macrolide antibiotic spiramycin (15 mg l^-1^), which partly retains its antibacterial activity at 60°C (Zorraquino et al., 2011), were performed as described in Zhang et al. (2015) together with serial dilutions ranging from 10^-5^ to 10^-8^ to obtain a highly enriched (∼85%) AOA culture that was used for further characterization.

Growth rates of “*Ca.* N. islandicus” were determined across a range of incubation temperatures (50–70°C). Triplicate cultures (25 ml) and negative controls (cultures not supplied with ammonium or inoculated with autoclaved biomass) were incubated for 10 days in 100 ml Schott bottles without agitation in the dark at the respective temperature. Samples from these experiments were either stored at -20°C for subsequent qPCR analyses (150 μl) or centrifuged (21,000 × *g*, 15 min, 18°C) to remove cells and the supernatant was stored at -20°C for chemical analysis (600 μl). qPCR analysis with primers CrenamoA19F (Leininger et al., 2006) and CrenamoA616R (Tourna et al., 2008) targeting the archaeal *amoA* gene was otherwise performed as described in Pjevac et al. (2017) before the genome sequence of “*Ca.* N. islandicus” was available. However, subsequent analysis demonstrated that the employed qPCR primers contain mismatches to the *amoA* sequence of this AOA in the middle of the forward and reverse primer. Average cell numbers were calculated from samples of triplicate cultures (*n* = 3 ± standard error). The specific growth rate was calculated from log-linear plots of *amoA* gene abundance in individual cultures. In this analysis, three out of seven time points were interpolated through linear regression.

To test whether the NO-scavenger 2-phenyl-4,4,5,5,-tetramethylimidazoline-3-oxide-1-oxyl (PTIO; TCI, Germany) inhibits ammonia oxidation by “*Ca.* N. islandicus,” 40 ml aliquots of mineral medium containing 1 mM ammonium were inoculated with 10% (v/v) of an exponential-phase culture and incubated in duplicates in the presence of 0, 33, and 100 μM PTIO, respectively. PTIO was dissolved in sterile mineral medium before addition to the cultures. The cultures not exposed to PTIO were supplemented with the same volume of sterile medium. The cultures were sampled (2 ml) at the beginning of the experiment and after 15 days of incubation. Nitrite and ammonium concentrations were measured as described above.

### DNA Extraction, Genome Sequencing, and Annotation

DNA from three replicate enrichment cultures containing “*Ca.* N. islandicus” as the only detectable ammonia oxidizer was extracted as described by Angel and Conrad (2013) and sequenced by Illumina HiSeq next generation sequencing (250 bp paired end reads). Since we did not obtain a complete genome with this approach we extracted genomic DNA from the enrichment at a later stage according to Zhou et al. (1996) yielding high molecular weight DNA. Genomic DNA was then sheared in a Covaris g-TUBE (Covaris, United States) at 9000 RPM for 2x 1 min. in an Eppendorf mini spin plus centrifuge (Eppendorf, DE). The DNA was run on a E-Gel EX 1% agarose gel (Thermo Fisher, United States) and small DNA fragments were removed by excising a band with a length of ∼8 kb. The DNA was purified from the gel cut using the ultraClean 15 DNA Purification Kit (Qiagen, United States). The DNA was prepared for sequencing using the “1D Low Input gDNA with PCR SQK-LSK108” protocol (Oxford Nanopore Technologies, United Kingdom) and sequenced on a FLO-MIN106 flowcell using the MinION MK1b (Oxford Nanopore Technologies, United Kingdom) following the manufacturers protocol using MinKNOW (v. 1.7.14). The nanopore reads were basecalled using Albacore (V. 2.0.1) (Oxford Nanopore Technologies, United Kingdom). The taxonomy was assigned to contigs as described in Karst et al. (unpublished) The read coverage was calculated from the read mappings. The complete genome was assembled using a hybrid approach combining the data from the Illumina and Nanopore sequencing with the hybrid assembler Unicycler (v. 0.4.1, Wick et al., 2017). The genome bins of the two contaminating organisms were assembled from the Nanopore reads using Miniasm (Li, 2016) and polished twice with the Nanopore reads using Racon (Vaser et al., 2017). No other microbe, encoding genes indicative for ammonia-oxidation, was identified in either of the two metagenomes.

The complete genome of “*Ca.* N. islandicus” was uploaded to the MicroScope platform (Vallenet et al., 2013) for automatic annotation, which was amended manually where necessary. The full genome sequence of “*Ca.* N. islandicus” has been deposited in GenBank (accession CP024014) and associated annotations are publicly available in MicroScope (“*Candidatus* Nitrosocaldus islandicus strain 3F”). The raw sequence (Illumina MiSeq and Nanopore) data has been deposited to ENA under Study accession number PRJEB24462.

Protein-coding genes from the thaumarchaeon studied here were compared to those from 30 Thaumarchaeota with available genomic data (Supplementary Table S1) downloaded from NCBI. The coding sequences (CDS) with accession numbers from each genome, as downloaded from NCBI, were combined with additional CDS predictions made by Prodigal (Hyatt et al., 2010) to account for variability in CDS predictions from different primary data providers and platforms. Predicted CDS from the thaumarchaeon of this study were aligned to CDS from reference genomes using blastp (Word_size = 2, substitution matrix BLOSUM45). Genes were considered homologous only if the blastp alignment exceeded 50% of the length of both query and subject sequences. CDS of “*Ca.* N. islandicus” that lacked any homologs in other Thaumarchaeota were considered “unique.” Unique CDS of unknown function were searched for secretion signals and for predicted membrane-spanning domains of the encoded proteins using the Phobius web server (Käll et al., 2007) and putative structures were determined using the Phyre2 web server (Kelley et al., 2015). Homology to “Thaumarchaeota-core” proteins was assessed by cross-referencing the blastp homology search to the proteins defined for “*Ca.* Nitrosotalea devanaterra” by Herbold et al. (2017).

### Phylogenetic Analysis and Habitat Preference

For 16S rRNA and *amoA* gene-based phylogenetic analysis, the full-length 16S rRNA and *amoA* gene sequences of “*Ca.* N. islandicus” retrieved from the genome assembly were imported into the ARB software package (Ludwig et al., 2004) together with other full length 16S rRNA or *amoA* gene sequences from cultivated AOA strains and aligned with the integrated ARB aligner with manual curation. 171 sequences from the Aigarchaea were included in the alignment and used as outgroup in the 16S rRNA gene phylogenetic analyses. For the *amoA* gene phylogenetic analyses no outgroup was selected. The 16S rRNA and *amoA* gene consensus trees were reconstructed using Maximum-Likelihood (ML; using the GTRGAMMA evolution model), Neighbour Joining (NJ) and Maximum Parsimony (MP) methods. For all calculations, a sequence filter considering only positions conserved in ≥50% of all thaumarchaeotal and aigarchaeal sequences was used, resulting in 2444 and 488 alignment positions for the 16S rRNA and *amoA* genes, respectively.

A Bayesian-inference phylogenomic tree was obtained using the automatically generated alignment of 34 concatenated universal marker genes (Supplementary Table S2), which were identified by CheckM in Parks et al. (2015). This alignment was used as input for PhyloBayes (Lartillot et al., 2009) with 10 independent chains of 4,000 generations using the CAT-GTR model; 2,000 generations of each chain were discarded as burn-in, the remainder were subsampled every second tree (bpcomp -x 2000 2 4000) and pooled together for calculation of posterior probabilities.

Whole-genome based average nucleotide identity (gANI, Varghese et al., 2015) and average amino acid identity values (AAI, Konstantinidis and Tiedje, 2005) were calculated between the genomes of “*Ca.* N. islandicus” and “*Ca.* N. yellowstonensis” using sets of annotated genes supplemented with additional gene calls predicted by Prodigal (Hyatt et al., 2010). gANI was calculated using the Microbial Species Identifier (MiSI) method (Varghese et al., 2015). For AAI, bidirectional best hits were identified using blastp, requiring that query genes aligned over at least 70% of their length to target genes (in each unidirectional blastp search). Query gene length was used to calculate a weighted average % identity over all best hit pairs and the calculations were repeated using each genome as query and target.

The occurrence of organisms closely related to “*Ca.* N. islandicus” and “*Ca.* N. yellowstonensis” in publicly deposited amplicon sequencing data sets was assessed using IMNGS (Lagkouvardos et al., 2016) with the full-length 16S rRNA gene sequences of both organisms as query and a nucleotide identity threshold of 97%.

*PolB* amino acid sequences were extracted from the arCOG database [arCOG14^1^ (arCOG15272, arCOG00329, arCOG00328, arCOG04926, arCOG15270)]. Additional thaumarchaeotal *polB* sequences were identified using “*Ca.* N. islandicus” as a query in a blastp search against the nr protein database. These additional thaumarchaeotal sequences, the *polB* sequence from “*Ca.* N. islandicus” and arCOG database sequences were de-replicated using usearch (Edgar, 2010) with -sortbylength and -cluster_smallmem (-id 0.99 -query_cov 0.9), aligned using default settings in mafft (Katoh and Standley, 2013) and a phylogenetic tree was calculated using FastTree (Price et al., 2010).

Nitrilase superfamily amino acid sequences were obtained from Pace and Brenner (2001). Alignment and phylogenetic reconstruction was carried out with Bali-Phy (Suchard and Redelings, 2006; randomize alignment, iterations = 11000, burnin = 6000). Posterior tree pools from 10 independent runs were combined to generate a 50% PP consensus tree and to assess bipartition support.

A dataset for assessing the phylogenetic relationship of the alpha subunit of 2-oxoacid:ferredoxin oxidoreductases (OFORs) was based on Gibson et al. (2016) and supplemented with additional indolepyruvate oxidoreductase (*ior*) sequences. Genomes available (as of October 30, 2017) from the NCBI genomes database were downloaded, genes were predicted using Prodigal V2.6.3 (Hyatt et al., 2010) and predicted genes were screened for *iorA* (TIGRFAM03336) using hmmsearch v3.1b2^2^ with an *e*-value cutoff of 0.001. Genes meeting the search criteria were used as queries against the complete TIGRFAM database to ensure that the extracted *iorA* sequences matched the *iorA* model as the best-hit model with an *e*-value cutoff of 0.001. Reciprocal best-hit genes were required to align to the hmm over at least 500 contiguous bases. Amino acid sequences were then clustered into centroids using usearch v8.0.1517 (sortbylength and cluster_smallmem -id 0.8 -query_cov 0.9; Edgar, 2010). Centroids were aligned using mafft v7.245 (Katoh and Standley, 2013) and trees were constructed using FastTree 2.1.4 (Price et al., 2010). The initial phylogenetic placement of “*Ca.* N. islandicus” *iorA* in the resulting large tree (3,179 sequences) was used to choose a small set of bacterial *iorA* sequences to include in the final tree. The final dataset was aligned using mafft v7.245 (Katoh and Standley, 2013) and trees were constructed using FastTree 2.1.4 (Price et al., 2010).

### Electron Microscopy

For scanning electron microscopy, “*Ca.* N. islandicus” cells were harvested by centrifugation (4,500 × *g*, 15 min, 25°C) and fixed on poly-L-lysine coated slides with a filter-sterilized 2.5% glutaraldehyde fixation solution in phosphate buffered saline (PBS; 130 mM NaCl in 5% [v/v] phosphate buffer mixture [20–80 v/v] of 200 mM NaH_2_PO_4_ and 200 mM Na_2_HPO_4_). Subsequently, fixed cells were washed three times for 10 min in PBS and post-fixed with a 1% OsO_4_ solution in PBS for 40 min. The fixed cells were again washed three times in PBS, dehydrated in a 30–100% (v/v) ethanol series, washed in acetone, and critical point dried with a CPD 300 unit (Leica). Samples were mounted on stubs, sputter coated with gold using a sputter coater JFC-2300HR (JEOL), and images were obtained with a JSM-IT300 scanning electron microscope (JEOL).

## Results and Discussion

### Enrichment and Basic Physiology of “*Ca.* N. islandicus”

**FIGURE 1:**
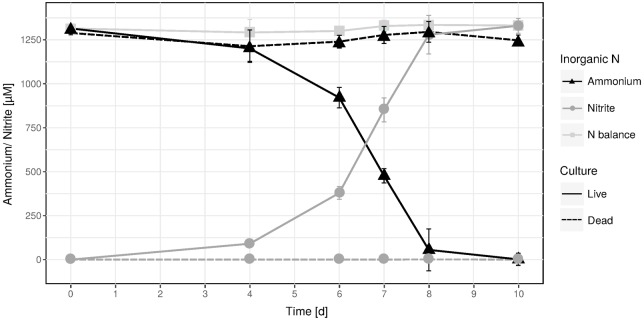
Near-stoichiometric oxidation of 1.25 mM ammonium to nitrite by the “*Ca.* N. islandicus” enrichment culture at 65°C. Data points show means, error bars show 1 SD of *n* = 3 biological replicates. Solid and dashed lines denote live and dead culture incubations, respectively. If not visible, error bars are smaller than symbols.

An ammonia-oxidizing enrichment culture was established from biofilm material sampled from a hot spring located in the geothermal valley Graendalur of South-Western Iceland. Temperature tests for optimal activity and growth were performed at different time points during the enrichment period and showed varying results, but below 50°C and above 75°C activity and growth was never observed. Only during the initial enrichment phase did ammonia oxidation occur at 75°C. At 65°C the highest ammonia oxidation rates and the shortest lag phases were usually measured (data not shown), however, in a single experiment the optimal temperature was 70°C (Supplementary Figure S1). Likely, these variations reflect varying abundance ratios of “*Ca.* N. islandicus” and accompanying bacteria over time as described in Lebedeva et al. (2008). A high enrichment level of a single AOA phylotype (see below) was achieved by applying the antibiotic spiramycin (15 mg l^-1^) followed by biomass transfers into fresh medium using serial dilutions. This enrichment culture showed near stoichiometric conversion of ammonium to nitrite when incubated at 65°C (**Figure 1**). This was accompanied by growth of the AOA with a specific growth rate of 0.128 ± 0.011 d^-1^ (mean generation time of 2.32 ± 0.24 d), which is substantially slower than those reported for “*Ca.* Nitrosocaldus yellowstonensis HL72,” *Nitrososphaera viennensis* EN76, or *Nitrosopumilus maritimus* SCM1 (Könneke et al., 2005; de la Torre et al., 2008; Martens-Habbena et al., 2009; Stieglmeier et al., 2014b; **Table 1**), but faster than a marine enrichment culture (Berg et al., 2015). At late exponential phase “*Ca*. N. islandicus” grown at 65°C reached a density of 1.90 × 10^4^ ± 2.84 × 10^3^ cells per μl.

**Table 1:** Genome features and growth rates of “*Candidatus* N. islandicus” and of selected reference ammonia-oxidizing archaea (AOA).

Genome features	“*Ca.* N. islandicus 3F”	*N. gargensis* Ga9-2	*N. viennensis* EN76	“*Ca.* N. exaquare G61”	“*Ca.* N. devanaterra Nd1”	“*Ca.* N. uzonensis N4”	*N. maritimus* SCM1
Genome size [Mb]	1.62	2.83	2.53	2.99	1.81	1.65	1.60
Number of scaffolds	1	1	1	1	1	1	1
Number of contigs	1	1	1	1	1	14	1
Average G+C content [%]	41.54	48.35	52.72	33.94	37.07	42.25	34.17
Protein coding density [%]	87.85	83.37	86.43	77.14	90.55	90.42	91.65
Number of genomic objects (CDS, fragment CDS, r/tRNA)	1851	4037	3266	3394	2145	2001	2012
Number of coding sequences (CDS)	1824	3999	3277	3358	2106	1960	1969
Motility/chemotaxis	+	+	+	-	+	+	-
Carbon fixation	3HP/4HB	3HP/4HB	3HP/4HB	3HP/4HB	3HP/4HB	3HP/4HB	3HP/4HB
Ammonium transporters	3	3	3	1	3	2	2
NirK	0	1	1	1	1	1	1
MCO1 + ZIP/MCO1^a^	1/0	1/1	2/0	1/1	0/0	1/0	2/0
Urease and urea transport	+	+	+	+	-	-	-
Cyanate lyase	-	+	-	-	-	-	-
Nitrilase/Cyanide hydratase	1	0	0	0	0	1	1
Aromatic amino acid fermentation	+	-	-	-	-	-	-
Hydrogenase	3b	-	4a	-	4a	-	-
Coenzyme F420	+	+	+	+	+	+	+
Vitamin B12	+	+	+	+	+	+	+
Catalase	0	(1)^b^	0	1	0	0	0
Peroxidase	0	0	0	1	0	0	0
Superoxide dismutase	1	1	1	1	1	1	2
Chlorite dismutase-like enzyme^c^	1	1	1	1	1	1	1
DNA polymerases	B1, Y	B1, D, Y	B1, D, Y	B1, D, Y	B1, D, Y	B1, D, Y	B1, D, Y
Generation time [d]	2.32 ± 0.24^d^	NA	1.25 ± 0.03	NA	NA	NA	0.88 – 1.08


*^*a*^MCO1+ZIP/MCO1, multicopper oxidase 1 (as defined in Kerou et al., 2016) with adjacent zinc permease/multicopper oxidase 1 without an adjacent zinc permease; ^*b*^The gene is truncated; ^*c*^chlorite dismutases are of interest in other nitrifiers (Maixner et al., 2008; Kostan et al., 2010), but it is not known what their function is in archaea; ^*d*^determined at 65°C; NA, not available.*

### Genome Reconstruction, Phylogeny, and Environmental Distribution

**FIGURE 2:**
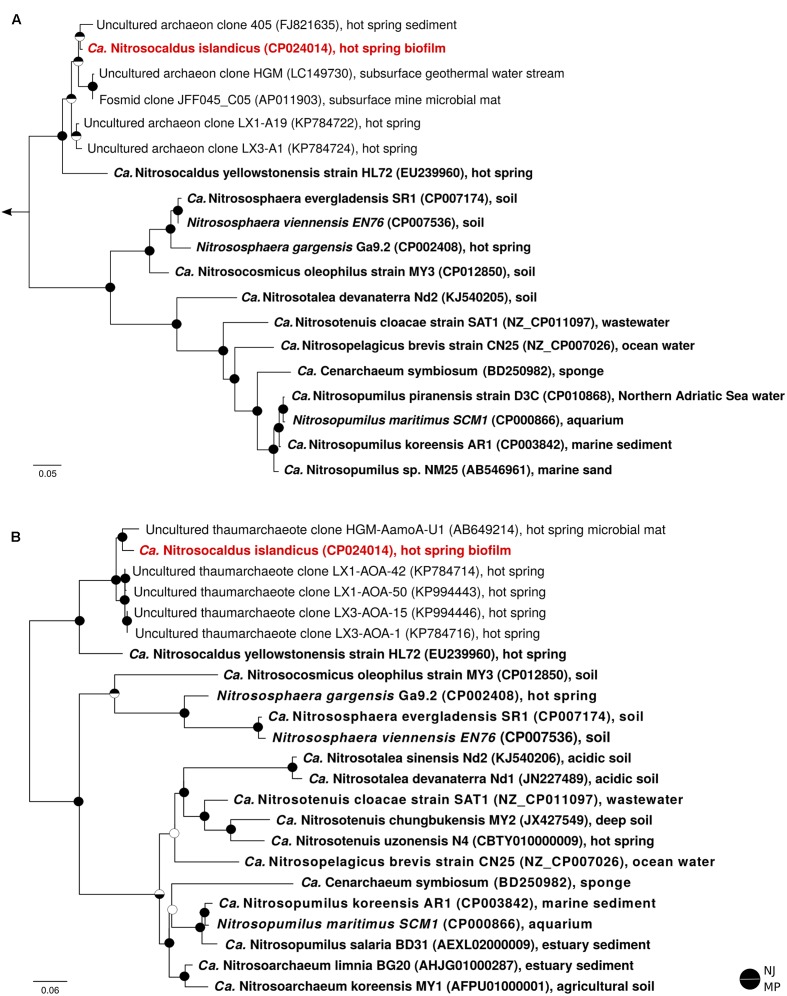
16S rRNA gene-based **(A)** and *amoA* gene-based **(B)** maximum likelihood phylogenies of representative thaumarchaeotal sequences. For each sequence, the accession number and environmental source are indicated. Sequences from pure and enrichment cultures are depicted in bold, and “*Ca*. N. islandicus” is highlighted in red. The outgroup for the 16S rRNA tree were aigarchaeal sequences; the *amoA* phylogeny was calculated unrooted, but artificially rooted to the Nitrosocaldales afterwards. Circles at nodes denote support (filled) or no support (open) from Neighbour Joining (NJ, top half) and Maximum Parsimony (MP, bottom half) trees. The scale bars in **(A,B)** indicate 9 and 6% sequence divergence, respectively.

**FIGURE 3:**
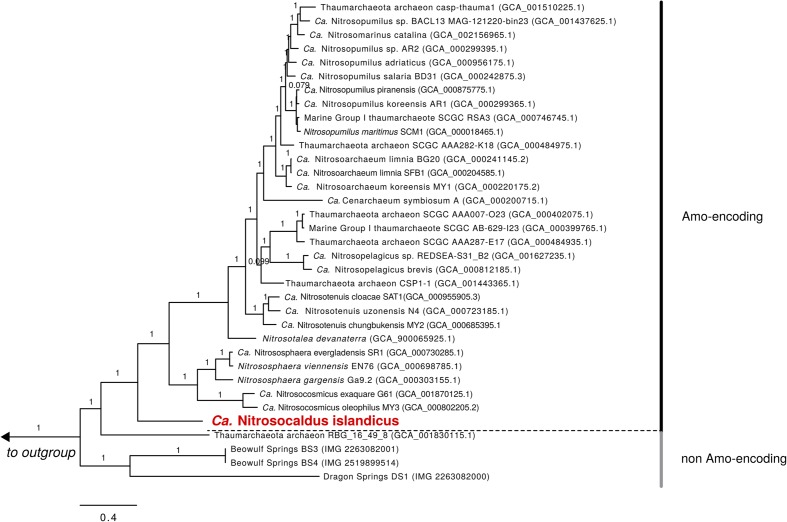
Bayesian inference tree of 34 concatenated universal marker proteins from 31 *amoA*-encoding Thaumarchaeota including the *Nitrosocaldus*-like AOA and 4 non-*amoA*-encoding Thaumarchaeota-like Archaea. Nineteen additional TACK-superphylum (Guy and Ettema, 2011) members (not shown) were used as an outgroup: Aigarchaea (assemblies GCA_000494145.1, GCA_000270325.1), Bathyarchaea (GCA_001399805.1, GCA_001399795.1, GCA_001593865.1, GCA_001593855.1, GCA_001593935.1, GCA_002011035.1, GCA_001273385.1), Crenarchaea (GCA_000011205.1, GCA_000591035.1, GCA_000253055.1, GCA_000813245.1), Geothermarchaea (GCA_002011075.1), Korarchaea (GCA_000019605.1), Thorarchaea (GCA_001563335.1, GCA_001563325.1), and Verstraetearchaea (GCA_001717035.1, GCA_001717015.1). Branches are labeled with average Bayesian posterior probability support over 10 independent chains and the scale bar indicates 0.4 amino acid substitutions per site.

Metagenomic sequencing of the enrichment culture with Illumina and Nanopore demonstrated that the current culture contained an AOA as the only taxon encoding the repertoire genes required for ammonia oxidation. Hybrid assembly allowed reconstruction of the complete genome of this AOA as one circular contiguous sequence of 1.62 Mbps length (**Table 1**). The 16S rRNA gene and *amoA* gene of the newly enriched AOA are 96 and 85% identical, respectively, to the genes of “*Ca.* Nitrosocaldus yellowstonensis,” the only other cultured obligately thermophilic AOA. The average amino acid sequence identity (AAI) and the genomic average nucleotide identity (gANI) between the genome and the one of “*Ca.* N. yellowstonensis” are 65.4% (alignment fraction: 0.86) and 75.8% (alignment fraction: 0.59), respectively, which is above the proposed genus and below the proposed species boundary thresholds (Qin et al., 2014; Varghese et al., 2015). Consequently, the enriched obligately thermophilic AOA was assigned to the same genus and referred to as “*Ca.* Nitrosocaldus islandicus.” According to 16S rRNA gene- based phylogenies, “*Ca.* N. islandicus” is a member of the Nitrosocaldales clade, which seems to predominantly encompass AOA from thermal environments (**Figure 2**). An extended phylogenomic analysis using a concatenated alignment of 34 proteins (Supplementary Table S2) identified by CheckM (Parks et al., 2015) confirmed that “*Ca.* N. islandicus” represents a basal lineage within the known ammonia-oxidizing Thaumarchaeota (**Figure 3**). This result lends strong support to the earlier notion, which was based on single-gene 16S rRNA and *amoA* phylogenies (de la Torre et al., 2008), that the thermophilic Nitrosocaldales clade is an early diverging group of the ammonia-oxidizing Thaumarchaeota. It would also be compatible with the possibility that archaeal ammonia oxidation originated in thermal environments (de la Torre et al., 2008; Hatzenpichler et al., 2008; Groussin and Gouy, 2011; Brochier-Armanet et al., 2012).

**FIGURE 4:**
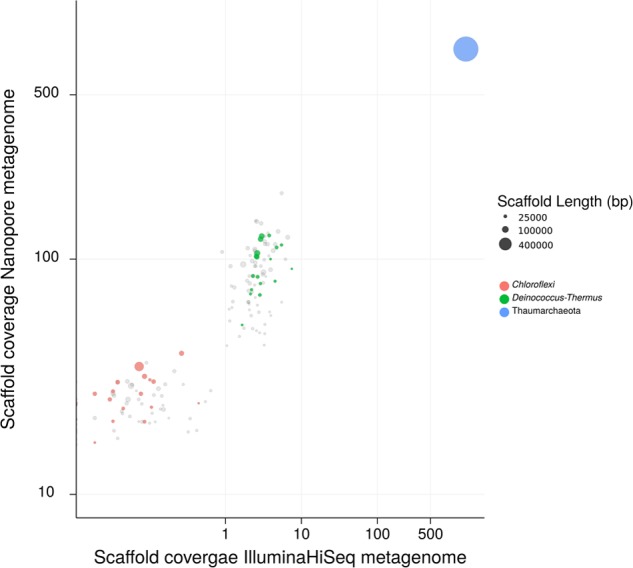
Sequence composition-independent binning of the metagenome scaffolds from two ammonia-oxidizing enrichment cultures. Circles represent scaffolds, scaled by the square root of their length. Clusters of similarly colored circles represent potential genome bins. The *x*-axis shows binning of the scaffolds from an early enrichment culture, which still included other genera as well (not shown). The *y*-axis shows binning of the scaffolds from the latest enrichment culture containing only “*Ca.* N. islandicus” and the two remaining accompanying organisms. Genome bins for the *Thermus* (34% complete) and the *Chloroflexi* (56% complete) organism were obtained. The genome bin of the *Chloroflexi* organism contains genes that cluster within a clade of *Nitrobacter*/*Nitrolancea* nitrite oxidoreductase (*nxrAB*) genes (data not shown). Some nitrate production by the batch enrichment culture was observed after multiple re-feedings with NH_4_^+^ (>20 mM). As NOB in the phylum Chloroflexi are known (Sorokin et al., 2012), it is tempting to speculate that this Chloroflexi may be a thermophilic nitrite oxidizer.

**FIGURE 5:**
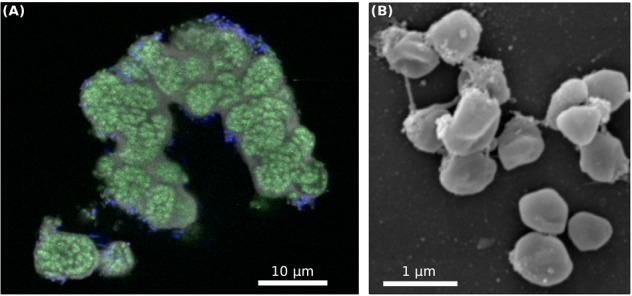
**(A)** FISH analysis of the enrichment culture illustrating the growth in microcolonies and the high relative abundance of “*Ca.* N. islandicus.” “*Ca.* N. islandicus” cells appear in green (stained by probe Thaum726 targeting most Thaumarchaeota) and the bacterial contaminants in blue (labeled by probe EUB338). **(B)** Scanning electron micrograph of spherically shaped “*Ca.* Nitrosocaldus islandicus” cells. The cells have a diameter of 0.5–0.7 μm. “*Ca.* N. islandicus” cells were distinguishable from the rod-shaped bacterial contaminants by their smaller size and unique, ‘dented’ spherical shape.

Metagenomic sequencing revealed that in addition to “*Ca.* N. islandicus” the culture also contained two heterotrophic bacterial contaminants, which were identified as a *Thermus* sp. and a member of the Chloroflexi phylum (**Figure 4**). The enrichment level of “*Ca.* N. islandicus” was approximately 85% based on read counts from the Nanopore sequencing, whereas the *Thermus* sp. and Chloroflexi accounted for 12 and 3%, respectively. FISH-analysis of the enrichment culture confirmed the dominance of “*Ca.* N. islandicus” and showed that the AOA grew mainly in aggregates, whereas the bacterial cells grew either co-localized with the archaeal flocs or planktonically (**Figure 5A**). Electron microscopy demonstrated that the cells of “*Ca.* N. islandicus” are small (with a diameter of approximately 0.5–0.7 μm) and have an irregular coccoid shape (**Figure 5B**). Morphologically they resemble the cells of “*Ca.* N. yellowstonensis” (Qin et al., 2017b).

**FIGURE 6:**
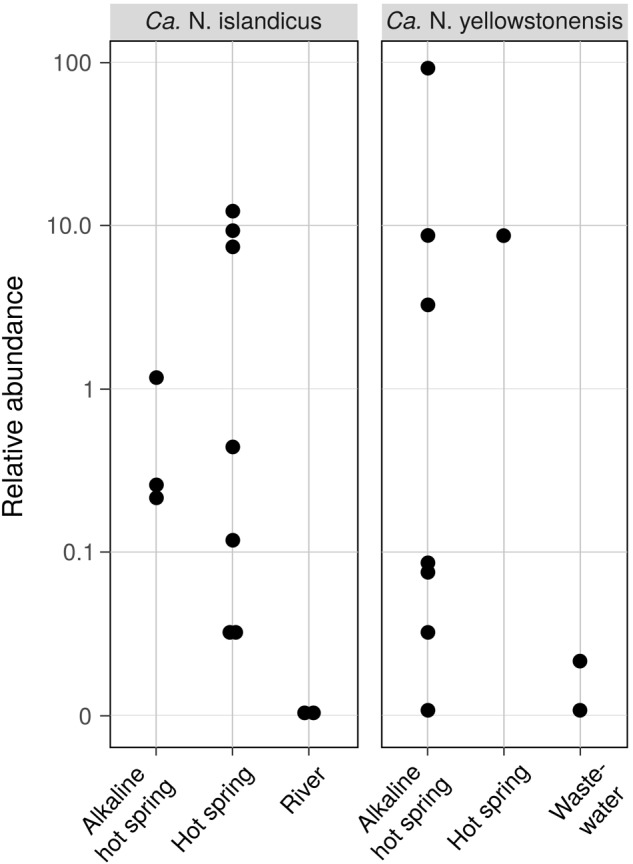
Occurrence and abundance of AOA related to “*Ca.* Nitrosocaldus islandicus” and “*Ca.* Nitrosocaldus yellowstonensis” in different habitats based on the presence of closely related 16S rRNA gene sequences in all public 16S rRNA gene amplicon datasets. Data shown are log-scale relative abundances of 16S rRNA gene sequences with a minimum similarity of 97% in a sample (*n* = 12 and *n* = 10 out of 93,045 total datasets for “*Ca.* N. islandicus” and “*Ca.* N. yellowstonensis,” respectively). Sequences of the Tibetan waste water data set where retrieved from BioSample SAMN03464927 of Niu et al. (2017).

The environmental distribution of the two cultured Nitrosocaldales members and closely related AOA was assessed by screening all publicly available 16S rRNA gene amplicon datasets (*n* = 93,045) for sequences highly similar (97%) to the 16S rRNA genes of “*Ca.* N. islandicus” and “*Ca.* N. yellowstonensis” using the pipeline described by Lagkouvardos et al. (2016). This analysis revealed that these taxa are highly confined in their distribution and occur predominantly in terrestrial hot springs where they can reach high relative abundances between 11.4 and 86% (“*Ca.* N. islandicus” and “*Ca.* N. yellowstonensis,” respectively) of the total microbial community (**Figure 6**). Interestingly, “*Ca.* N. yellowstonensis”-related organisms seem to occur mainly in hot springs described as alkaline with a pH of around 8.5, but were also detected in a sample from a Tibetan wastewater treatment plant (Niu et al., 2017). The unexpected detection of members of the Nitrosocaldales in the latter sample was confirmed by 16S rRNA-based phylogenetic analyses (data not shown) and it would be interesting to know whether this wastewater treatment plant is in some way connected to water from a close-by hot spring.

### Genome Features

Addition of the complete genome of “*Ca.* N. islandicus” to the set of available thaumarchaeotal genome sequences (*n* = 30) reduced the number of gene families identified as representing the “Thaumarchaeota-core” (Herbold et al., 2017) from 743 to 669 (reduction by 9.96%; Supplementary Table S3). In a few cases, genes with low sequence homology to apparently absent core gene families are actually present in the genome of “*Ca.* N. islandicus,” but were not scored as they did not match the alignment length criterion. For example, “*Ca.* N. islandicus,” like all other AOA sequenced to date, has a gene encoding the K-subunit of RNA polymerase class I, but with a low sequence similarity to the respective orthologous genes in other AOA. In a few other cases, enzymes found in all other AOA genomes are absent but functionally replaced by members of different enzyme families. For example, all other genome-sequenced AOA contain a cobalamin-dependent methionine synthase. In contrast, “*Ca.* N. islandicus” possesses only an unrelated cobalamin-independent methionine synthase, which is also found in some other AOAs.

In addition to updating the thaumarchaeotal core genome we also specifically looked for genes that are present in “*Ca.* N. islandicus,” but were not reported for other AOA before. In the following sections, the most interesting findings from these analyses are reported and put in context.

**FIGURE 7:**
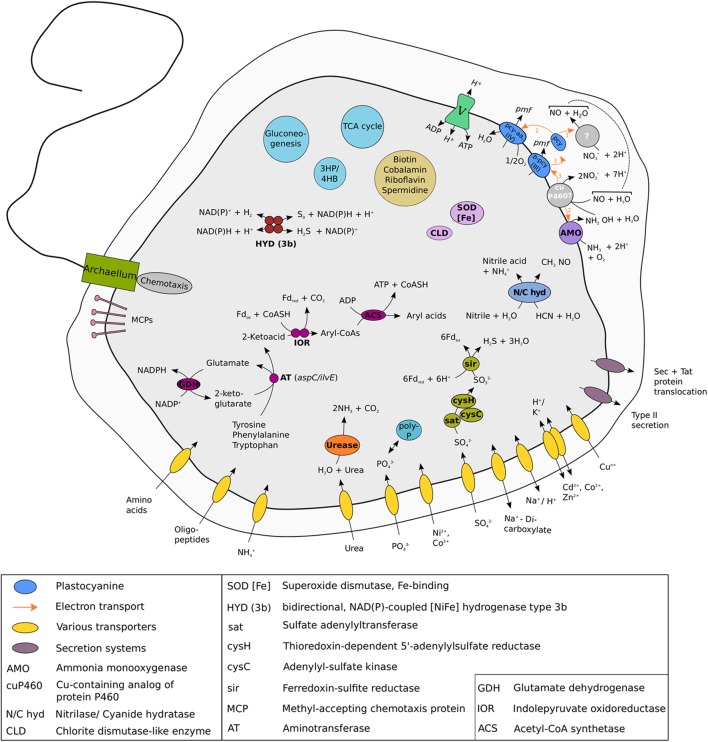
Cell metabolic cartoon constructed from the annotation of the “*Ca.* N. islandicus” genome. Enzyme complexes of the electron transport chain are labeled by Roman numerals. Most metabolic features displayed are discussed in the main text. The model of ammonia oxidation and electron transfer is depicted as proposed by Kozlowski et al. (2016). Locus tags of all genes discussed in the main text are given in Supplementary Table S4. Like some but not all AOA, “*Ca.* N. islandicus” encodes all genes required to assemble an archaeal flagellar apparatus that is composed of the flagellar filament, the motor, and its switch (Mosier et al., 2012; Spang et al., 2012; Lebedeva et al., 2013; Lehtovirta-Morley et al., 2016; Li, 2016; Qin et al., 2017a), although no archaellum could be detected in our electron microscopic analysis. The *fla* gene cluster of “*Ca.* N. islandicus” shows a similar arrangement to *Nitrososphaera gargensis* and contains six genes including one gene for structural flagellin subunit FlaB/FlaA as well as the flagellar accessory genes *flaG*, *flaF*, *flaH*, *flaJ*, and *flaI* (Spang et al., 2012).

Like all other AOA, the “*Ca.* N. islandicus” genome encodes the typical repertoire for CO_2_ fixation via the modified 3-hydroxypropionate/4-hydroxybutyrate (3HP/4HB) cycle and for archaeal ammonia oxidation (**Figure 7**, **Table 1** and Supplementary Figure S2) (Walker et al., 2010; Spang et al., 2012; Könneke et al., 2014; Otte et al., 2015; Kerou et al., 2016). Unexpectedly, however, the gene *nirK* encoding an NO-forming nitrite reductase (NirK) is absent. NirK has been suggested to play an essential role for ammonia oxidation in AOA by providing NO for the NO-dependent dehydrogenation of hydroxylamine to nitrite (Kozlowski et al., 2016). Interestingly, ammonia oxidation by “*Ca.* N. islandicus” was completely inhibited after the addition of ≥33 μM of the NO-scavenger PTIO (Supplementary Figure S3), a concentration that is lower or in the same range as previously reported to be inhibitory for other AOA (Shen et al., 2013; Jung et al., 2014; Martens-Habbena et al., 2015; Sauder et al., 2016). This finding suggests that NO is required for ammonia oxidation in “*Ca.* N. islandicus” despite the absence of NirK. The only other known AOA without a *nirK* gene are the sponge symbiont “*Ca.* Cenarchaeum symbiosum” (Hallam et al., 2006; Bartossek et al., 2010) and “*Ca.* N. yellowstonensis” (Stahl and de la Torre, 2012). For the uncultured “*Ca.* C. symbiosum” ammonia-oxidizing activity has not been demonstrated and the absence of *nirK* might have resulted from gene loss during adaptation to a life-style as symbiont. “*Ca.* N. yellowstonensis” is the closest cultured representative of “*Ca.* N. islandicus,” and the lack of *nirK* may thus be a common feature of the Nitrosocaldales. These AOA might produce NO by a yet unknown mechanism. In this context it is interesting to note that the hydroxylamine dehydrogenase of AOB, of which the functional homolog in archaea has not been identified yet, has recently been reported to produce NO instead of nitrite (Caranto and Lancaster, 2017). Alternatively, NO could be provided by accompanying organisms such as the *Thermus* and Chloroflexi-like bacteria that remain in the enrichment. Indeed, the genome bins obtained for these organisms both encode a *nirK* gene. The *Thermus sp.* genome bin further contains a *norBC* and *narGH* genes, in line with described denitrification capabilities for the genus *Thermus* (Alvarez et al., 2014). A dependence of Nitrosocaldales on NO production by other microorganisms could explain why no pure culture from this lineage has been obtained yet.

“*Ca.* N. islandicus” possesses genes coding for urease that are present in some but not all AOA (Walker et al., 2010; Spang et al., 2012; Kerou et al., 2016; Lehtovirta-Morley et al., 2016; Sauder et al., 2017) (**Figure 7** and **Table 1**), but lacks a cyanase that is used by *Nitrososphaera gargensis* for cyanate-based growth (Palatinszky et al., 2015). Additionally, the genome encodes an enzyme that either belongs to a novel class of the nitrilase superfamily or to the cyanide hydratase family (**Figure 7** and Supplementary Figure S4). Nitrilases catalyze the direct cleavage of a nitrile to the corresponding acid while forming ammonia (Pace and Brenner, 2001) and cyanide hydratases convert HCN to formamide. Both substrates are relatively thermostable (Isidorov et al., 1992; Miyakawa et al., 2002). Nitriles occur as intermediates of microbial metabolism (Kobayashi et al., 1993) and nitrile hydratases have previously been isolated from several thermophiles (Cramp et al., 1997; Almatawah et al., 1999; Kabaivanova et al., 2008). Furthermore, both compounds are intermediates of the proposed abiotic synthesis of organics at hydrothermal sites (Miller and Urey, 1959; Schulte and Shock, 1995) and could thus be available in the hot spring habitat of “*Ca.* N. islandicus.” Similar genes have been found in the genomes of several other AOA from the *Nitrosopumilus* and *Nitrosotenuis* genera (Walker et al., 2010; Mosier et al., 2012; Lebedeva et al., 2013; Park et al., 2014; Bayer et al., 2016) (**Table 1**) and it will be interesting to find out for which metabolism they may be used in AOA.

Intriguingly, “*Ca.* N. islandicus” might be able to ferment amino acids under anaerobic conditions as it contains the entire pathway used by some hyperthermophilic archaea for ATP generation from aromatic amino acids (Mai and Adams, 1994; Adams et al., 2001; Ozawa et al., 2012) (**Figure 7**). In this pathway arylpyruvates are formed from aromatic amino acids by the activity of amino acid aminotransferases using 2-oxoglutarate as the amine group acceptor. The glutamate produced by this transamination can be recycled back to 2-oxoglutarate via glutamate dehydrogenase (*gdhA*) with the concomitant reduction of NADP^+^. With *ilvE* and *aspC* genes present, “*Ca.* N. islandicus” encodes at least two enzymes for which an aminotransferase activity specific for tyrosine, phenylalanine and aspartate has been demonstrated (Gelfand and Steinberg, 1977). Subsequently, these 2-ketoacids could be oxidatively decarboxylated and converted to aryl-CoAs by the oxygen-sensitive enzyme indolepyruvate oxidoreductase (Ozawa et al., 2012) encoded by *iorAB* using oxidized ferredoxin as electron acceptor. *IorAB* is absent from all other genome-sequenced AOA and does also not occur in the ancestral thaumarchaeote “FN1” (Lin et al., 2015) that lacks AMO. The *ior* genes present in “*Ca.* N. islandicus” have the highest similarity to and cluster together with *iorAB*-genes found in *Kyrpidia tusciae* and Dadabacteria (Supplementary Figure S5). Finally, transformation of aryl-CoAs to aryl acids catalyzed by ADP-dependent acetyl-CoA/acyl-CoA synthetase (Glasemacher et al., 1997) leads to ATP formation via substrate-level phosphorylation (**Figure 7**). “*Ca.* N. islandicus” encodes four acetyl-CoA/acyl-CoA synthetases, two of which are most similar to non-syntenous homologs of acetyl-CoA/acyl-CoA synthetases found in other AOA. However, the third gene is absent in all other AOA to date and its most similar homologs are encoded by species of the peptidolysing thermophilic archaea *Thermoproteus* and *Sulfolobus* and the fourth is most similar to an acetyl-/acyl-CoA synthetase found in members of the thermophilic Bathyarchaea and Hadesarchaea.

The fermentation of aromatic amino acids also requires regeneration of oxidized ferredoxin (reduced by IorAB) and NADP^+^ (reduced by glutamate dehydrogenase). However, no canonical ferredoxin:NADP^+^ oxidoreductase, or other enzymes (Buckel and Thauer, 2013) described to regenerate oxidized ferredoxin, are encoded in the genome of “*Ca.* N. islandicus.” It seems unlikely that the amount of ferredoxin oxidized by an encoded ferredoxin-dependent assimilatory sulfite/nitrite reductase (**Figure 7**) would be sufficient to compensate for all ferredoxin reduced in the dissimilatory fermentation pathway. However, “*Ca*. N. islandicus” can also oxidize reduced ferredoxin with a 2:oxoglutarate-ferredoxin oxidoreductase (Supplementary Figure S5). NAD(P)H can be re-oxidized by a cytosolic, bidirectional, NAD(P)-coupled type 3b [NiFe] –hydrogenase that is encoded by “*Ca.* N. islandicus” in contrast to all other genomically characterized AOA (**Figure 7** and **Table 1**). NAD(P)H oxidation by this hydrogenase could lead to hydrogen generation, or the enzyme could act as a sulfhydrogenase that reduces zero valent sulfur compounds (produced by other organisms or present in the environment) to hydrogen sulfide (Ma et al., 1993; Adams et al., 2001). The hydrogenase genes are clustered at a single locus and code for the four subunits of the holoenzyme and accessory proteins (Supplementary Figure S6). This hydrogenase might also allow “*Ca.* N. islandicus” to use hydrogen as energy source providing reduced NAD(P)H under oxic conditions as this type of hydrogenase has been shown to tolerate exposure to oxygen (Bryant and Adams, 1989; Berney et al., 2014; Kwan et al., 2015).

Surprisingly, the genome of “*Ca.* N. islandicus” lacks genes for both subunits of the DNA polymerase D (PolD), which is present in all other AOA and most archaeal lineages (including thermophiles) with the exception of the Crenarchaea (Cann et al., 1998; Makarova et al., 2014; Saw et al., 2015) (**Table 1**). It is assumed that either PolD alone or together with DNA polymerases of the B family (PolB) is required for DNA synthesis and elongation in these archaea (Cubonová et al., 2013; Ishino and Ishino, 2013; Makarova et al., 2014). The “*Ca.* N. islandicus” genome encodes only one B-type DNA polymerase (PolB1, Supplementary Figure S7) and one DNA polymerase of the Y family (PolY), generally considered to be involved in the rescue of stalled replication forks and enhancement of cell survival upon DNA damage (Friedberg et al., 2002). Recently, it has been demonstrated for the PolD-lacking crenarchaeon *Sulfolobus acidocaldarius* that both its PolB1 and PolY have polymerase activities *in vitro* (Peng et al., 2016). However, “*Ca.* N. islandicus” (like other AOA) does not encode the PolB1-binding proteins PBP1 and PBP2, which are required to form a multisubunit DNA polymerase holoenzyme together with PolB in the crenarchaeon *S. solfataricus* P2 (Yan et al., 2017). We hypothesize that “*Ca.* N. islandicus” may utilize one or both of the present polymerases for DNA replication, possibly in combination with its heterodimer PriSL, which has been demonstrated to function as a primase, a terminal transferase and a polymerase capable of polymerizing RNA or DNA chains of up to 7,000 nucleotides (Lao-Sirieix and Bell, 2004).

It is also interesting to note that the obligate thermophile “*Ca.* N. islandicus” like all genome-sequenced Thaumarchaeota (Spang et al., 2017) does not encode a reverse gyrase, which is widespread in hyperthermophilic microbes including other archaea of the TACK superphylum (Makarova et al., 2007; Heine and Chandra, 2009; López-García et al., 2015), but is not essential for growth under these conditions (Atomi et al., 2004).

## Conclusion

We have obtained a highly enriched (∼85%) culture of an obligately thermophilic AOA from a hot spring in Iceland. Despite the impressive diversity of AOA in high temperature environments as revealed by molecular tools (Zhang et al., 2008; Wang et al., 2009; Zhao et al., 2011; Nishizawa et al., 2013; Li et al., 2015; Chen et al., 2016), cultivation of only a single obligately thermophilic AOA species – “*Ca.* Nitrosocaldus yellowstonensis” – was reported before (de la Torre et al., 2008). The newly enriched AOA represents a new species of the genus *Nitrosocaldus* and was named “*Ca.* N. islandicus.” Comparative analysis of its closed genome revealed several surprising features like the absence of DNA polymerase D and the lack of canonical NO-generating enzymes^§^, although physiological experiments with a NO-scavenger demonstrated NO-dependent ammonia-oxidation, as described for other AOA (Shen et al., 2013; Jung et al., 2014; Martens-Habbena et al., 2015; Sauder et al., 2016). Furthermore, “*Ca.* N. islandicus” encodes the enzymatic repertoire for fermentation of aromatic amino acids that is, so far, unique among sequenced AOA. A pure culture of “*Ca.* N. islandicus” will be required to physiologically verify this genome-based hypothesis. Peptide or aromatic amino acid fermentation would enable an anaerobic lifestyle of “*Ca.* N. islandicus” and, if more widespread among Thaumarchaeota not yet characterized (including mesophiles), might help explain their sometimes surprisingly high abundance in anaerobic ecosystems (Molina et al., 2010; Bouskill et al., 2012; Buckles et al., 2013; Beam et al., 2014; Lin et al., 2015).

Based on the data presented here, we propose the following provisional taxonomic assignment for the thaumarchaeon in our enrichment culture.

Nitrosocaldales order

Nitrosocaldaceae fam.

‘*Candidatus* Nitrosocaldus islandicus’ sp. nov.

### Etymology

Nitrosus (Latin masculine adjective): nitrous; caldus (Latin masculine adjective): hot; islandicus (Latin masculine genitive name): from Iceland. The name alludes to the physiology of the organism (ammonia oxidizer, thermophilic) and the habitat from which it was recovered.

### Locality

The biofilm of a terrestrial hot spring in Graendalur geothermal valley, Iceland (64° 1′7″ N, 21° 11′20″ W).

### Diagnosis

An obligately thermophilic, aerobic chemolithoautotrophic ammonia oxidizer from the phylum Thaumarchaeota growing as small irregular shaped cocci. The values of AAI and gANI between this species and its closest cultured relative, “*Ca.* N. yellowstonensis”, are 65.4 and 75.8%, respectively.

## Note Added in Proof

After submission of this manuscript a second report on the enrichment and genome analysis of a thermophilic thaumarchaeote was submitted and accepted for publication in *Frontiers in Microbiology* (doi: 10.3389/fmicb.2018.00028). The genomes of both organisms share many of the above discussed features such as the absence of *nirK* and genes encoding polymerase D, and thereby lend support to the possibility that these are common genomic features of the Nitrosocaldales.

## Author Contributions

AD, JV, and CS cultivated and enriched the culture. AD, CS, and PP performed growth and activity experiments. AD performed FISH and SEM analysis. CH, AD, PP, MA, and RK performed bioinformatic analysis. JdlT kindly provided access to the “*Ca.* N. yellowstonensis” genome. AD, JV, CS, PP, MW, and HD manually curated the annotation of the genome and interpreted the genome data. AD and MW wrote the manuscript with help from all co-authors.

## Conflict of Interest Statement

MA and RK own and run DNASense, the sequencing center at which the metagenomes were sequenced and the bins assembled. The other authors declare that the research was conducted in the absence of any commercial or financial relationships that could be construed as a potential conflict of interest.

## Footnotes

1 ftp://ftp.ncbi.nih.gov/pub/wolf/COGs/arCOG/

2 http://hmmer.org

§ See section “Note Added in Proof”.
