# Supplementary material for: Cultivation and Genomic Analysis of “Candidatus Nitrosocaldus islandicus,” an Obligately Thermophilic, Ammonia-Oxidizing Thaumarchaeon from a Hot Spring Biofilm in Graendalur Valley, Iceland
Source: Front Microbiol. 2018 Feb 14;9:193. doi: 10.3389/fmicb.2018.00193 (PMC5817080; doi:10.3389/fmicb.2018.00193)
Supplement: Supplementary file 1 [file Presentation_1.pdf]

Supplemental information

Table S1. Thaumarchaeota used for comparison of protein coding genes

Organism Name	Publication	BioSample	BioProject	Assembly
Thaumarchaeota archaeon casp-thauma1 (Caspean Sea)	10.7717/peerj.2687	SAMN03733542	PRJNA279271	GCA_001510225.1
Nitrosopumilus sp. BACL13 MAG-121220-bin23	10.1186/s13059-015-0834-7	SAMN03741946	PRJNA273799	GCA_001437625.1
Ca. Nitrosomarinus catalina	10.1111/1462-2920.13768	SAMN05730076	PRJNA341864	GCA_002156965.1
Ca. Nitrosopumilus sp. AR2	10.1128/JB.01869-12	SAMN02603138	PRJNA174388	GCA_000299395.1
Ca. Nitrosopumilus adriaticus	10.1038/ismej.2015.200	SAMN03253153	PRJNA269341	GCA_000956175.1
Ca. Nitrosopumilus salaria BD31	10.1128/JB.00013-12.	SAMN00016669	PRJNA50075	GCA_000242875.3
Ca. Nitrosopumilus piranensis	10.1038/ismej.2015.200	SAMN03257648	PRJNA269924	GCA_000875775.1
Ca. Nitrosopumilus koreensis AR1	10.1128/JB.01857-12	SAMN02603137	PRJNA174387	GCA_000299365.1
Marine Group I thaumarchaeote SCGC RSA3 (Red Sea)	10.1038/ismej.2014.137	SAMN02869648	PRJNA248555	GCA_000746745.1
Nitrosopumilus maritimus SCM1	10.1073/pnas.0913533107	SAMN00000032	PRJNA19265	GCA_000018465.1
Thaumarchaeota archaeon SCGC AAA282-K18	10.3389/fmicb.2016.00143	SAMN02440765	PRJNA190793	GCA_000484975.1
Ca. Nitrosoarchaeum limnia BG20	10.1128/JB.00007-12.	SAMN00016663	PRJNA50027	GCA_000241145.2
Ca. Nitrosoarchaeum limnia SFB1	10.1371/journal.pone.0016626.	SAMN02471010	PRJNA52465	GCA_000204585.1
Ca. Nitrosoarchaeum koreensis MY1	10.1128/JB.05717-11.	SAMN02470178	PRJNA67913	GCA_000220175.2
Ca. Cenarchaeum symbiosum A	10.1073/pnas.0608549103	SAMN02744041	PRJNA202	GCA_000200715.1
Thaumarchaeota archaeon SCGC AAA007-O23	10.3389/fmicb.2016.00143	SAMN02440520	PRJNA66857	GCA_000402075.1
Marine Group I thaumarchaeote SCGC AB-629-I23	10.1038/ismej.2014.137	SAMN02441296	PRJNA165501	GCA_000399765.1
Thaumarchaeota archaeon SCGC AAA287-E17	10.3389/fmicb.2016.00143	SAMN02441105	PRJNA190806	GCA_000484935.1
Nitrosopelagicus sp. REDSEA-S31_B2	10.1038/sdata.2016.50, PMC: 4932879	SAMN04534603	PRJNA289734	GCA_001627235.1
Ca. Nitrosopelagicus brevis	10.1073/pnas.1416223112	SAMN03273964	PRJNA223412	GCA_000812185.1
Thaumarchaeota archaeon CSP1-1 (sediment)	10.1111/1462-2920.12930	SAMN03462092	PRJNA262935	GCA_001443365.1
Ca. Nitrosotenuis cloacae	10.1038/srep23747	SAMN03286947	PRJNA272771	GCA_000955905.3
Ca. Nitrosotenuis uzonensis N4	10.1371/journal.pone.0080835	SAMEA3139018	PRJEB4650	GCA_000723185.1
Ca. Nitrosotenuis chungbukensis MY2	10.1128/AEM.03730-13	SAMN02767256	PRJNA210247	GCA_000685395.1
Ca. Nitrosotalea devanaterra	10.1128/AEM.04031-15	SAMEA3577360	PRJEB10948	GCA_900065925.1
Ca. Nitrososphaera evergladensis SR1	10.1371/journal.pone.0101648	SAMN03081530	PRJNA235208	GCA_000730285.1
Nitrososphaera viennensis EN76	10.1073/pnas.1601212113	SAMN02721150	PRJEA60103	GCA_000698785.1
Nitrososphaera gargensis Ga9.2	10.1111/j.1462-2920.2012.02893.x	SAMN02603264	PRJNA60505	GCA_000303155.1
Ca. Nitrosocosmicus exaquare G61	10.1038/ismej.2016.192	SAMN04606696	PRJNA317395	GCA_001870125.1
Ca. Nitrosocosmicus oleophilus MY3	10.1111/1758-2229.12477	SAMN03074222	PRJNA210256	GCA_000802205.2

Table S2. Marker genes used for phylogenomic tree			
**Gene**	**Pfam Id**	**Length**	**Description**
Alanine – tRNA ligase	TIGR00344	847	Alanine – tRNA ligase
Ribosomal protein L10	PF00466	100	Ribosomal protein L10
Ribosomal protein L11	PF03946	60	Ribosomal protein L11, N-terminal domain
Ribosomal protein L11	PF00298	69	Ribosomal protein L11, RNA binding domain
Ribosomal protein L13	PF00572	128	Ribosomal protein L13
Ribosomal protein L14p/L23e	PF00238	122	Ribosomal protein L14p/L23e
Ribosomal protein L16p/L10e	PF00252	133	Ribosomal protein L16p/L10e
Ribosomal protein L18p/L5e	PF00861	119	Ribosomal protein L18p/L5e
Ribosomal protein L1p/L10e	PF00687	220	Ribosomal protein L1p/L10e
Ribosomal protein L22p/L17e	PF00237	105	Ribosomal protein L22p/L17e
Ribosomal protein L23	PF00276	92	Ribosomal protein L23
Ribosomal protein L29	PF00831	58	Ribosomal protein L29
Ribosomal protein L3	PF00297	263	Ribosomal protein L3
Ribosomal protein L4/L1	PF00573	192	Ribosomal protein L4/L1
Ribosomal protein L5	PF00281	56	Ribosomal protein L5
Ribosomal protein L5	PF00673	95	Ribosomal protein L5P, C-terminus
Ribosomal protein S11	PF00411	110	Ribosomal protein S11
Ribosomal protein S12/S23	PF00164	122	Ribosomal protein S12/S23
Ribosomal protein S15	PF00312	83	Ribosomal protein S15
Ribosomal protein S17	PF00366	69	Ribosomal protein S17
Ribosomal protein S19	PF00203	81	Ribosomal protein S19
Ribosomal protein S2	PF00318	211	Ribosomal protein S2
Ribosomal protein S3	PF00189	85	Ribosomal protein S3, C-terminal domain
Ribosomal protein S5	PF03719	74	Ribosomal protein S5, C-terminal domain
Ribosomal protein S5	PF00333	67	Ribosomal protein S5, N-terminal domain
Ribosomal protein S7p/S5e	PF00177	148	Ribosomal protein S7p/S5e
Ribosomal protein S8	PF00410	129	Ribosomal protein S8
Ribosomal protein S9/S16	PF00380	121	Ribosomal protein S9/S16
Ribosomal Protein L2	PF03947	130	Ribosomal Proteins L2, C-terminal domain
Ribosomal protein L2	PF00181	77	Ribosomal proteins L2, RNA binding domain
RNA polymerase beta subunit	PF04563	203	RNA polymerase beta subunit
RNA polymerase Rpb1	PF04997	337	RNA polymerase Rpb1, domain 1
RNA polymerase Rpb1	PF00623	166	RNA polymerase Rpb1, domain 2
RNA polymerase Rpb1	PF05000	108	RNA polymerase Rpb1, domain 4
RNA polymerase Rpb2	PF04561	190	RNA polymerase Rpb2, domain 2
RNA polymerase Rpb2	PF04565	68	RNA polymerase Rpb2, domain 3
RNA polymerase Rpb2	PF00562	386	RNA polymerase Rpb2, domain 6
RNA polymerase Rpb2	PF04560	82	RNA polymerase Rpb2, domain 7
RNA polymerase Rpb6	PF01192	57	RNA polymerase Rpb6
Signal peptide binding domain	PF02978	104	Signal peptide binding domain
Translation-initiation factor 2	PF11987	109	Translation-initiation factor 2
TruB family pseudouridylate synthase	PF01509	149	TruB family pseudouridylate synthase
Valine – tRNA ligase	TIGR00422	863	Valine – tRNA ligase

Table S3. Genes not present in “Ca. N. islandicus”, but previously present in the “Thaumarchaeota-core” as defined by[27]

Locus tag of “core” gene in Ca. N. devanaterra	Annotation
NDEV_v3_0007|ID:23877774|	DNA polymerase II large subunit
NDEV_v3_0191|ID:23877958|	Protein pelota homolog
NDEV_v3_0273|ID:23878040|	SAM-dependent methyltransferase
NDEV_v3_0276|ID:23878043|	5-carboxymethyl-2-hydroxymuconate Delta-isomerase
NDEV_v3_0416|ID:23878183|	methionine sulfoxide reductase B
NDEV_v3_0872|ID:23878639|	UvrABC system protein C
NDEV_v3_0873|ID:23878640|	ATPase and DNA damage recognition protein of nucleotide excision repair excinuclease UvrABC
NDEV_v3_0874|ID:23878641|	excinulease of nucleotide excision repair, DNA damage recognition component
NDEV_v3_0889|ID:23878656|	Iron-containing alcohol dehydrogenase
NDEV_v3_0897|ID:23878664|	Endoribonuclease L-PSP
NDEV_v3_0915|ID:23878682|	Double-stranded beta-helix fold enzyme
NDEV_v3_0916|ID:23878683|	putative methyltransferase type 11
NDEV_v3_0925|ID:23878692|	Transcriptional regulator, ArsR family
NDEV_v3_0952|ID:23878719|	Disulfide Bond oxidoreductase D family protein
NDEV_v3_1056|ID:23878823|	Rossmann fold nucleotide-binding protein
NDEV_v3_1068|ID:23878835|	CMP/dCMP deaminase zinc-binding
NDEV_v3_1143|ID:23878910|	Oligoendopeptidase, PepF/M3 family
NDEV_v3_1165|ID:23878932|	Pyruvoyl-dependent arginine decarboxylase
NDEV_v3_1181|ID:23878948|	putative SMC domain protein
NDEV_v3_1198|ID:23878965|	Elongation factor Tu domain 2 protein
NDEV_v3_1303|ID:23879070|	Peptide methionine sulfoxide reductase MsrA
NDEV_v3_1775|ID:23879542|	Modification methylase LlaDCHIA
NDEV_v3_1856|ID:23879623|	putative bacterial transferase hexapeptide (Three repeats)
NDEV_v3_1858|ID:23879625|	Glycosyl transferase family protein
NDEV_v3_2071|ID:23879838|	Peptidyl-prolyl cis-trans isomerase
NDEV_v3_2075|ID:23879842|	PfkB domain protein
NDEV_v3_2078|ID:23879845|	Putative pyridoxal phosphate-dependent aminotransferase
NDEV_v3_2106|ID:23879873|	DNA-directed DNA polymerase
NDEV_v3_0018|ID:23877785|	conserved protein of unknown function
NDEV_v3_0103|ID:23877870|	Uncharacterized membrane protein required for N-linked glycosylation (Modular protein)
NDEV_v3_0319|ID:23878086|	conserved protein of unknown function
NDEV_v3_0351|ID:23878118|	Membrane protein-like protein
NDEV_v3_0419|ID:23878186|	conserved protein of unknown function
NDEV_v3_0557|ID:23878324|	conserved protein of unknown function
NDEV_v3_0732|ID:23878499|	conserved protein of unknown function
NDEV_v3_0831|ID:23878598|	conserved protein of unknown function
NDEV_v3_0848|ID:23878615|	conserved protein of unknown function
NDEV_v3_1197|ID:23878964|	conserved protein of unknown function
NDEV_v3_1246|ID:23879013|	conserved membrane protein of unknown function
NDEV_v3_1619|ID:23879386|	protein of unknown function
NDEV_v3_1649|ID:23879416|	protein of unknown function
NDEV_v3_1654|ID:23879421|	conserved protein of unknown function
NDEV_v3_1755|ID:23879522|	protein of unknown function
NDEV_v3_1759|ID:23879526|	conserved protein of unknown function
NDEV_v3_1776|ID:23879543|	protein of unknown function
NDEV_v3_1831|ID:23879598|	protein of unknown function
NDEV_v3_1852|ID:23879619|	conserved protein of unknown function
NDEV_v3_1910|ID:23879677|	Conserved protein of unknown function
NDEV_v3_1936|ID:23879703|	conserved exported protein of unknown function
NDEV_v3_2039|ID:23879806|	protein of unknown function
NDEV_v3_0100|ID:23877867|	Putative nucleic acid binding protein
NDEV_v3_0101|ID:23877868|	Putative nucleic acid binding protein
NDEV_v3_0267|ID:23878034|	protein of unknown function
NDEV_v3_0428|ID:23878195|	exported protein of unknown function
NDEV_v3_0951|ID:23878718|	Redoxin domain protein
NDEV_v3_1248|ID:23879015|	Methyltransferase type 11
NDEV_v3_1377|ID:23879144|	conserved membrane protein of unknown function
NDEV_v3_1561|ID:23879328|	conserved protein of unknown function
NDEV_v3_1613|ID:23879380|	Methionine synthase
NDEV_v3_1614|ID:23879381|	Homocysteine S-methyltransferase
NDEV_v3_1985|ID:23879752|	DNA topoisomerase type IA zn finger domain protein
NDEV_v3_0126|ID:23877893|	ABC transporter, permease component
NDEV_v3_1837|ID:23879604|	conserved protein of unknown function
NDEV_v3_0184|ID:23877951|	DEAD/DEAH box helicase domain protein
NDEV_v3_0023|ID:23877790|	CCA-adding enzyme
NDEV_v3_0924|ID:23878691|	Phosphoribosylaminoimidazole-succinocarboxamide synthase
NDEV_v3_0004|ID:23877771|	conserved protein of unknown function
NDEV_v3_0522|ID:23878289|	Molecular chaperone
NDEV_v3_1051|ID:23878818|	conserved protein of unknown function
NDEV_v3_0413|ID:23878180|	4Fe-4S ferredoxin iron-sulfur binding domain protein
NDEV_v3_1807|ID:23879574|	conserved protein of unknown function
NDEV_v3_0792|ID:23878559|	RNA polymerase Rpb6
NDEV_v3_0699|ID:23878466|	Uncharacterized Zn-finger containing protein
NDEV_v3_1725|ID:23879492|	ThiamineS protein

Table S4. Genome locus tags and annotations of genes discussed in the main text.

Gene	Product	Locus tag
3-hydroxypropionate-4-hydroxybutyrate pathway		
accB/pccB	Acetyl-CoA/ propionyl-CoA carboxylase, carboxyltransferase subunit	AOA3F1_v2_0346
accC/pccC	acetyl-CoA carboxylase, biotin carboxylase subunit	AOA3F1_v2_0345
accA/pccA	biotin carboxyl carrier protein of put. acetyl-CoA/ propionyl-CoA carboxylase	AOA3F1_v2_0344
	putative methylmalonyl-CoA epimerase	AOA3F1_v2_0551
	methylmalonyl-CoA mutase, large subunit	AOA3F1_v2_0552
	methylmalonyl-CoA mutase, small subunit, C-terminus	AOA3F1_v2_1302
	4-hydroxybutyryl-CoA dehydratase	AOA3F1_v2_0024
	4-hydroxybutyryl-CoA dehydratase	AOA3F1_v2_0025
	3-hydroxybutyryl-CoA dehydratase	AOA3F1_v2_0462
	(S)-3-hydroxybutyryl-CoA dehydrogenase (NAD+)	
	Acetoacetyl-CoA thiolase or ketoacyl-CoA thiolase	
Candidate genes for 3-hydroxypropionate-4-hydroxybutyrate carbon fixation pathway including alcohol dehydrogenases		
asd	aspartate-semialdehyde dehydrogenase	AOA3F1_v2_0008
	3-hydroxyacyl-CoA dehydrogenase	AOA3F1_v2_1520
acs	Acetyl-coenzyme A synthetase	AOA3F1_v2_0573
acsA-1	Acetyl-coenzyme A synthetase	AOA3F1_v2_0981
acsA-2	Acetyl-coenzyme A synthetase	AOA3F1_v2_1057
gabD	succinate-semialdehyde dehydrogenase	AOA3F1_v2_0424
	Succinate-semialdehyde dehydrogenase (acetylating)	AOA3F1_v2_0975
	acetoacetyl-CoA thiolase or ketoacyl-CoA thiolase	AOA3F1_v2_0535
	acetoacetyl-CoA thiolase	AOA3F1_v2_0064
	putative CoA-binding protein	AOA3F1_v2_0829
	Protein with CoA-binding domain	AOA3F1_v2_0830
Tricarboxylic acid cycle		
gltA	citrate synthase	AOA3F1_v2_0976
acnA	aconitate hydratase	AOA3F1_v2_0442
	putative isocitrate/isopropylmalate dehydrogenase	AOA3F1_v2_1453
(o)orAC	2:oxoacid-ferredoxin oxidoreductase, fused alpha and gamma subunit	AOA3F1_v2_1720
(o)orB	2:oxoacid-ferredoxin oxidoreductase, beta subunit	AOA3F1_v2_1719
sucD	succinyl-CoA ligase, subunit alpha	AOA3F1_v2_1193
sucC	succinyl-CoA ligase, subunit beta	AOA3F1_v2_1192
sdhA	succinate dehydrogenase flavoprotein subunit/fumarate reductase	AOA3F1_v2_1407
(sdhC)	putative succinate dehydrogenase/fumarate reductase	AOA3F1_v2_1406
(sdhD)	putative succinate dehydrogenase/fumarate reductase	AOA3F1_v2_1405
sdhB	FeS-center protein of succinate dehydrogenase/fumarate reductase	AOA3F1_v2_1404
fumC	fumarate hydratase	AOA3F1_v2_0771
mdh	malate dehydrogenase	AOA3F1_v2_0339
ytsJ	NAD(P)-dependent malic enzyme	AOA3F1_v2_1553
Gluconeogenesis		
pckA	ATP-dependent phosphoenolpyruvate carboxykinase	AOA3F1_v2_0396
ppdk	pyruvate, phosphate dikinase	AOA3F1_v2_1141
eno	enolase	AOA3F1_v2_0437
apgM	2,3-bisphosphoglycerate-independent phosphoglycerate mutase	AOA3F1_v2_0723
(gpmB)	putative 2,3-bisphosphoglycerate-dependent phosphoglycerate mutase	AOA3F1_v2_0364
pgk	phosphoglycerate kinase	AOA3F1_v2_1746
(gap)	putative glyceraldehyde-3-phosphate dehydrogenase, phosphorylating	AOA3F1_v2_0467
tpiA	triosephosphate isomerase	AOA3F1_v2_1135
fbp	Inositol-1-monophosphatase/ bifunctional fructose-1,6-bisphosphatase	AOA3F1_v2_1480
11 subunit version of Complex I: type I NADH dehydrogenase (nuoEFG absent)		
nuoA	NADH-quinone oxidoreductase, subunit A	AOA3F1_v2_1711
nuoB	NADH-quinone oxidoreductase, subunit B	AOA3F1_v2_1710
nuoC	NADH-quinone oxidoreductase, subunit C	AOA3F1_v2_1709
nuoD	NADH-quinone oxidoreductase, subunit D	AOA3F1_v2_1708
nuoH	NADH-quinone oxidoreductase, subunit H	AOA3F1_v2_1707
nuoI	NADH-quinone oxidoreductase, subunit I	AOA3F1_v2_1706
nuoJ	NADH-quinone oxidoreductase, subunit J	AOA3F1_v2_1705
nuoK	NADH-quinone oxidoreductase, subunit K	AOA3F1_v2_1703
nuoM	NADH-quinone oxidoreductase, subunit M	AOA3F1_v2_1702
nuoL	NADH-quinone oxidoreductase, subunit L	AOA3F1_v2_1701
nuoN	NADH-quinone oxidoreductase, subunit N	AOA3F1_v2_1699

**Table S4** continued		
alternative NADH dehydrogenases, type-II NADH dehydrogenase, Coenzyme F420 dependent NADP dehydrogenase, nitroreductases, NADH-dependent FMN reductases, FAD dependent oxidoreductases		
	putative FAD-dependent pyridine nucleotide-disulphide oxidoreductase	AOA3F1_v2_0069
	putative NADH dehydrogenase/NAD(P)H nitroreductase AF_0226	AOA3F1_v2_1059
	FAD/NAD(P)-binding oxidoreductase	AOA3F1_v2_1072
	Geranylgeranyl reductase family protein	AOA3F1_v2_1423
Complex II		
sdhA	succinate dehydrogenase flavoprotein subunit/fumarate reductase	AOA3F1_v2_1407
(sdhC)	putative succinate dehydrogenase/fumarate reductase	AOA3F1_v2_1406
(sdhD)	putative succinate dehydrogenase/fumarate reductase	AOA3F1_v2_1405
sdhB	FeS-center protein of succinate dehydrogenase/fumarate reductase	AOA3F1_v2_1404
Complex III: 1.10.2.2		
petB	cytochrome b/b6 domain	AOA3F1_v2_1784
	Rieske iron sulfur protein	AOA3F1_v2_1785
	putative Rieske (2Fe-2S) domain protein	AOA3F1_v2_1749
Complex IV		
	conserved hypothetical protein	AOA3F1_v2_0365
(coxB)	putative heme-copper oxidase subunit II	AOA3F1_v2_0366
coxA	Cytochrome c oxidase polypeptide 1	AOA3F1_v2_0367
	blue (type 1) copper domain protein	AOA3F1_v2_0368
	putative Blue (Type 1) copper domain protein	AOA3F1_v2_0302
	putative cytochrome oxidase assembly protein	AOA3F1_v2_0369
Complex V: A1A0-type ATPase		
atpE	archaeal A1A0-type ATP synthase, subunit E	AOA3F1_v2_0087
atpA	archaeal A1A0-type ATP synthase, subunit A	AOA3F1_v2_0086
atpB	archaeal A1A0-type ATP synthase, subunit B	AOA3F1_v2_0085
atpD	archaeal A1A0-type ATP synthase, subunit D	AOA3F1_v2_0084
atpK	archaeal A1A0-type ATP synthase, subunit K	AOA3F1_v2_0082
atpI	archaeal A1A0-type ATP synthase, subunit I	AOA3F1_v2_0081
atpC	archaeal A1A0-type ATP synthase, subunit C	AOA3F1_v2_0080
atpF	archaeal A1A0-type ATP synthase, subunit F	AOA3F1_v2_0836
Electron carriers, plastocyanines		
	blue (type 1) copper domain protein	AOA3F1_v2_0368
	blue (type 1) copper domain protein	AOA3F1_v2_1174
	blue (type 1) copper domain protein	AOA3F1_v2_0302
	putative blue (type 1) copper domain protein	AOA3F1_v2_0302
	putative blue (type 1) copper domain protein	AOA3F1_v2_1780
	putative blue (type 1) copper domain protein (fragment)	AOA3F1_v2_1088
Multicopper oxigenases		
	multicopper oxidase type 3 (MCO1)	AOA3F1_v2_1374
Ammonia-monooxygenase		
amoB	putative archaeal ammonia monooxygenase subunit B	AOA3F1_v2_1440
amoC	Ammonia monooxygenase/methane monooxygenase, subunit C	AOA3F1_v2_1439
amoX	conserved hypothetical protein	AOA3F1_v2_1438
amoA	putative archaeal ammonia monooxygenase subunit A	AOA3F1_v2_1437
Urease		
ureD	Urease accessory protein UreD	AOA3F1_v2_0945
ureG	Urease accessory protein UreG	AOA3F1_v2_0944
ureF	Urease accessory protein UreF	AOA3F1_v2_0943
ureE	Urease accessory protein UreE	AOA3F1_v2_0942
ureC	Urease subunit alpha	AOA3F1_v2_0939
ureB	Urease subunit beta	AOA3F1_v2_0938
ureA	Urease gamma subunit	AOA3F1_v2_0937
Nitrilase/cyanide hydratase		
	putative Nitrilase/cyanide hydratase and apolipoprotein N-acyltransferase	AOA3F1_v2_0558
Detoxification		
(cld)	putative chlorite dismutase	AOA3F1_v2_1762
sod	Superoxide dismutase [Fe]	AOA3F1_v2_0392

**Table S4** continued		
Flagellum and Chemotaxis		
(flaK)	putative archaeal preflagellin peptidase FlaK	AOA3F1_v2_0613
flaB	archaeal flagellin	AOA3F1_v2_0578
	hypothetical protein	AOA3F1_v2_0579
flaG	flagellar protein FlaG	AOA3F1_v2_0580
flaF	flagellar protein FlaF	AOA3F1_v2_0581
flaH	flagella protein FlaH	AOA3F1_v2_0582
(flaJ)	putative flagella assembly protein FlaJ	AOA3F1_v2_0583
flaI	archaeal flagella protein FlaI	AOA3F1_v2_0584
(cheR)	putative chemotaxis MCP methyltransferase CheR	AOA3F1_v2_0585
cheD	chemoreceptor glutamine deamidase CheD	AOA3F1_v2_0586
(cheC)	putative chemotaxis protein CheC	AOA3F1_v2_0587
cheA	chemotactic sensor histidine kinase CheA	AOA3F1_v2_0594
cheB	chemotaxis response regulator methylesterase CheB	AOA3F1_v2_0593
cheY	chemotaxis response regulator CheY	AOA3F1_v2_0592
cheW	chemotaxis protein CheW	AOA3F1_v2_0591
(MCP)	putative methyl-accepting chemotaxis protein	AOA3F1_v2_0590
(MCP)	putative methyl-accepting chemotaxis protein	AOA3F1_v2_0589
	putative HEAT repeat-containing PBS lyase	AOA3F1_v2_0153
S assimilation		
(cysA)	putative thiosulfate sulfurtransferase	AOA3F1_v2_1414
sat	Sulfate adenylyltransferase	AOA3F1_v2_0054
(cysC)	putative adenylyl-sulfate kinase	AOA3F1_v2_1343
cysH	Thioredoxin-dependent 5'-adenylylsulfate reductase	AOA3F1_v2_0053
sir	Ferredoxin-sulfite/nitrite reductase	AOA3F1_v2_0949
	Rhodenase-Sulfurtransferase	AOA3F1_v2_0950
Amino acid fermentation		
ilvE	putative branched-chain-amino-acid aminotransferase	AOA3F1_v2_1069
ilvE-2	putative branched-chain-amino-acid aminotransferase	AOA3F1_v2_1492
aspC	Aspartate/ tyrosine/ aromtatic aminotransferase	AOA3F1_v2_1546
gdhA	Glutamate dehydrogenase	AOA3F1_v2_0468
iorB	Indolepyruvate oxidoreductase, beta subunit	AOA3F1_v2_1273
iorA	Indolepyruvate ferredoxin oxidoreductase, alpha subunit	AOA3F1_v2_1272
acs	Acetyl-coenzyme A synthetase	AOA3F1_v2_0573
acsA	Acetyl-coenzyme A synthetase	AOA3F1_v2_0981
acsA	Acetyl-coenzyme A synthetase	AOA3F1_v2_1057
	Acyl-CoA synthetase (NDP forming)	AOA3F1_v2_1415
sir	Ferredoxin-sulfite/nitrite reductase	AOA3F1_v2_0949
korB	2-oxoglutarate synthase subunit beta	AOA3F1_v2_1719
korA	2-oxoglutarate synthase subunit alpha	AOA3F1_v2_1720
Hydrogenase		
(hypA)	putative Hydrogenase nickel incorporation protein HypA	AOA3F1_v2_1388
	putative Hydrogenase 2 maturation protease	AOA3F1_v2_1387
(hydA)	Nickel-dependent hydrogenase alpha subunit	AOA3F1_v2_1386
(hydD)	putative Nickel-dependent hydrogenase delta subunit	AOA3F1_v2_1385
(hydG)	putative Nickel-dependent hydrogenase gamma subunit	AOA3F1_v2_1384
	putative Cyclic nucleotide-binding protein, hydrogenase accessory protein	AOA3F1_v2_1383
(hydB)	putative Nickel-dependent hydrogenase beta subunit	AOA3F1_v2_1382
hypF	putative carbamoyltransferase HypF	AOA3F1_v2_1380
(hypC/hupF)	putative Hydrogenase assembly chaperone HypC/HupF	AOA3F1_v2_1255
DNA replication and repair		
dbh2	DNA polymerase IV	AOA3F1_v2_0742
dpo	DNA polymerase B1	AOA3F1_v2_0645
	putative primase / polymerase	AOA3F1_v2_0930
priB	DNA primase large subunit PriL	AOA3F1_v2_0032
priA	putative DNA primase small subunit PriS	AOA3F1_v2_0030
dnaG	DNA primase DnaG	AOA3F1_v2_0952

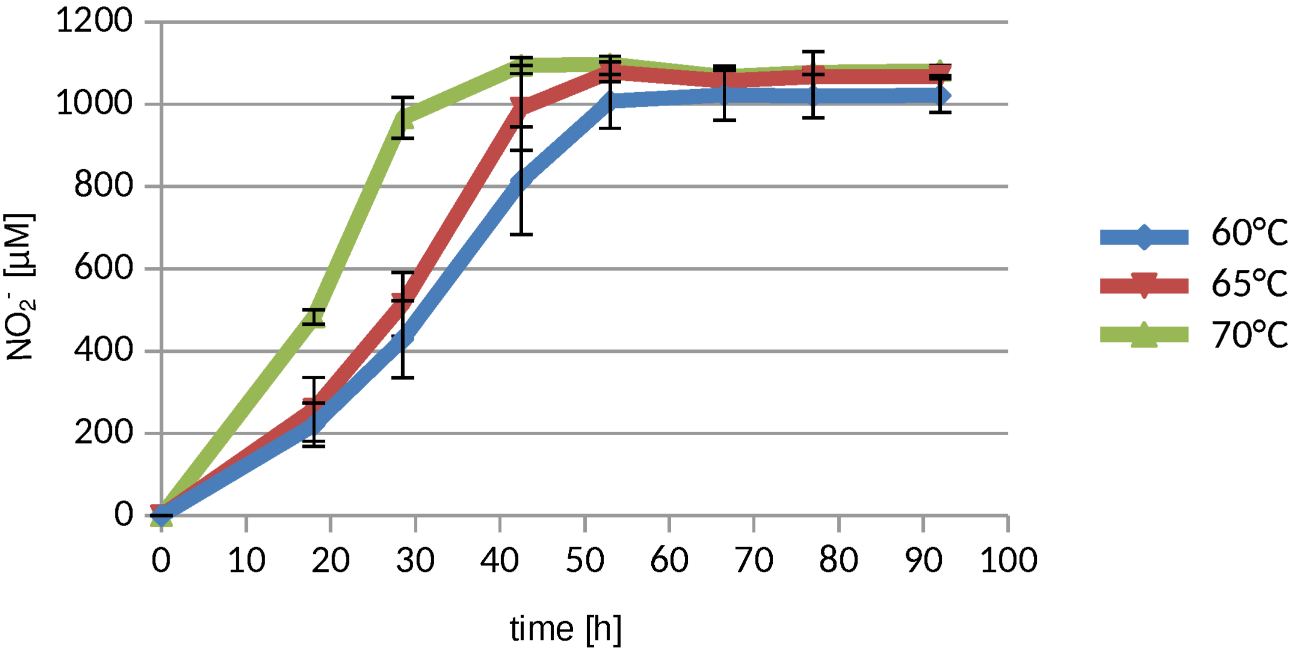

Figure S1. Nitrite accumulation through oxidation of ammonia at three different temperatures. Data points show means, error bars show standard errors of n = 3 biological replicates.

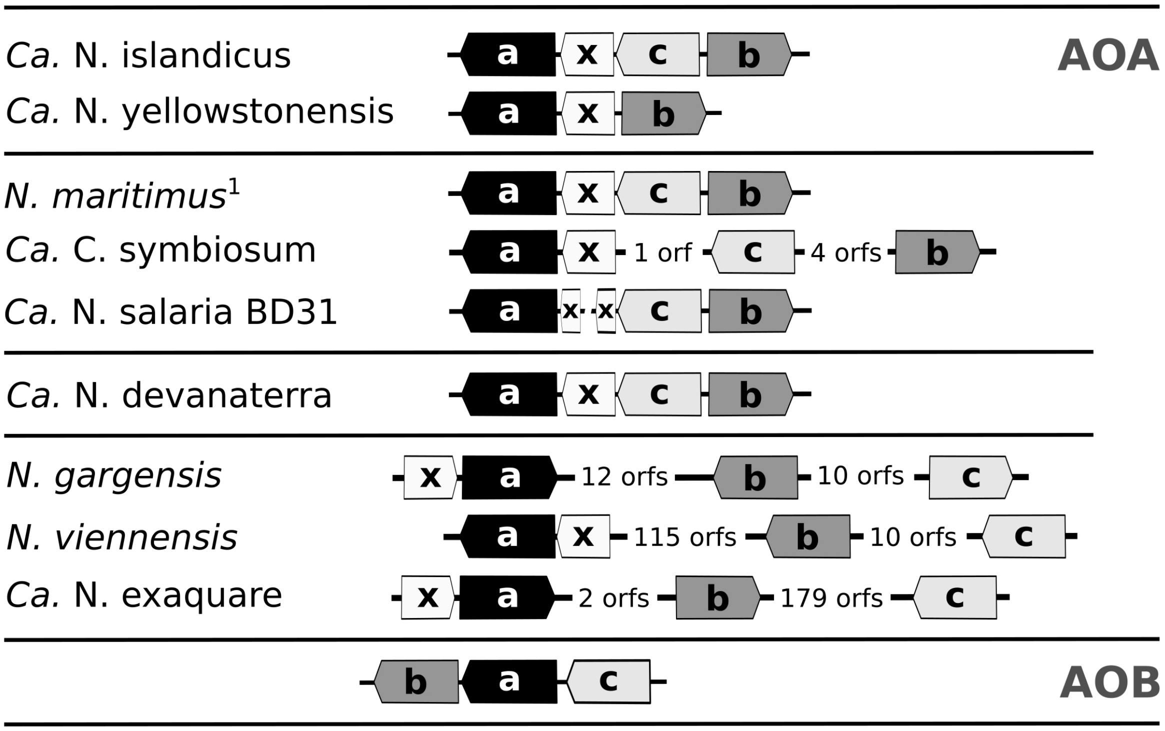

Figure S2. Gene order and orientation of the ammonia monooxygenase subunits (*amoA, amoB, amoC*, and the putative ‘‘*amoX*’’) in “*Ca.* N. islandicus” and other Thaumarchaeota. The gene order of ammonia-oxidizing bacteria (AOB) is given on the bottom as a reference. [1] also represents the gene arrangement in “*Ca.* N. limnia”, “*Ca.* N. koreensis” and “*Ca.* N. uzonensis”. The figure is a modified version of the figure 26.3 in (*28*)

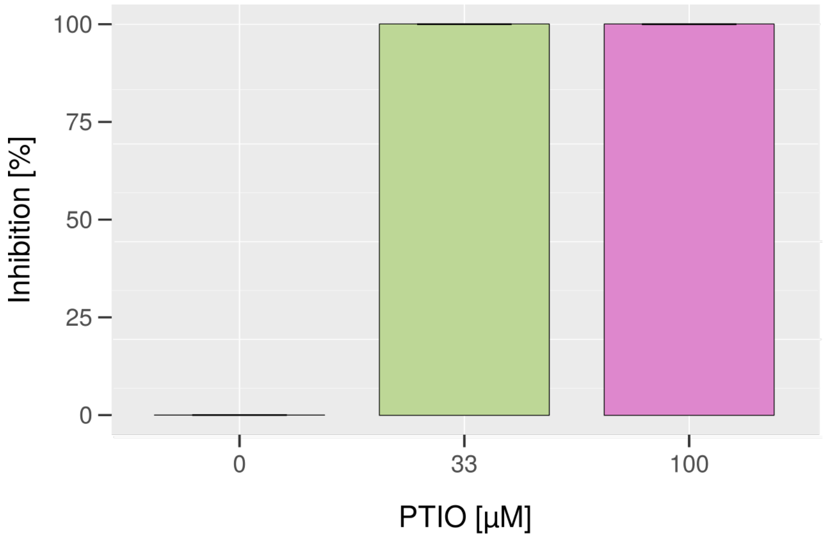

Figure S3. Inhibition of ammonia oxidation by "*Ca.* N. islandicus" caused by different concentrations of the NO-scavenger 2-phenyl-4,4,5,5-tetramethylimidazoline-1-oxyl 3-oxide (PTIO). Error bars show the standard error of two replicates.

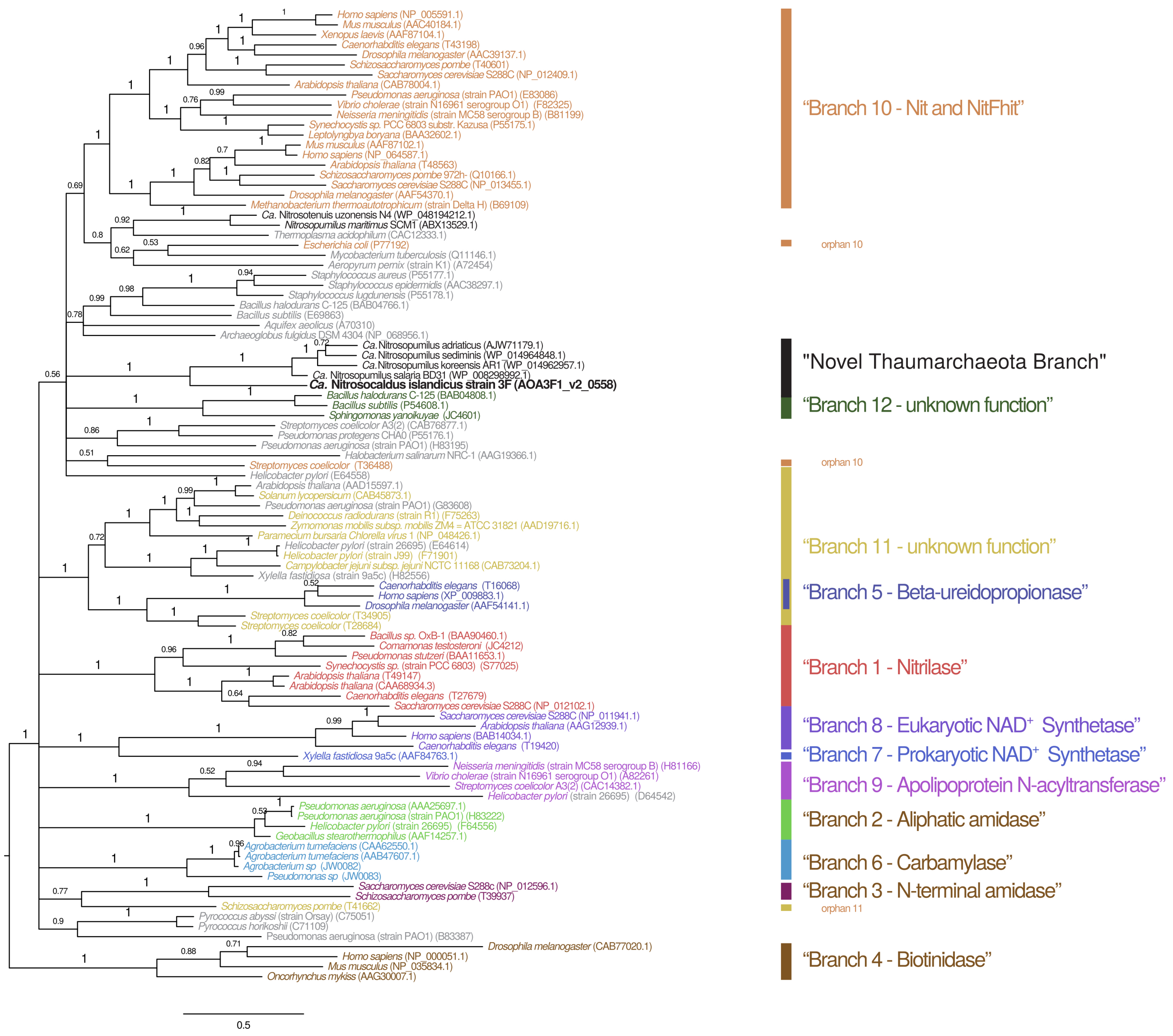

Figure S4. Phylogenetic tree of nitrilase superfamily members showing placement of "*Ca.* Nitrosocaldus islandicus strain 3F" in a novel branch consisting of Thaumarchaeota (Novel Thaumarchaeota branch). Sequences and branch labels are from (*29*). Grey labels indicate "nonfused outliers" as indicated by (*29)* which were not assigned to any of the 12 named branches. Black labels indicate sequences obtained from the thaumarchaeotal genomes. Sequences assigned to branches 10 and 11 in (*29)* that do not clade with other members of those branches in this phylogenetic tree are labelled "orphan".

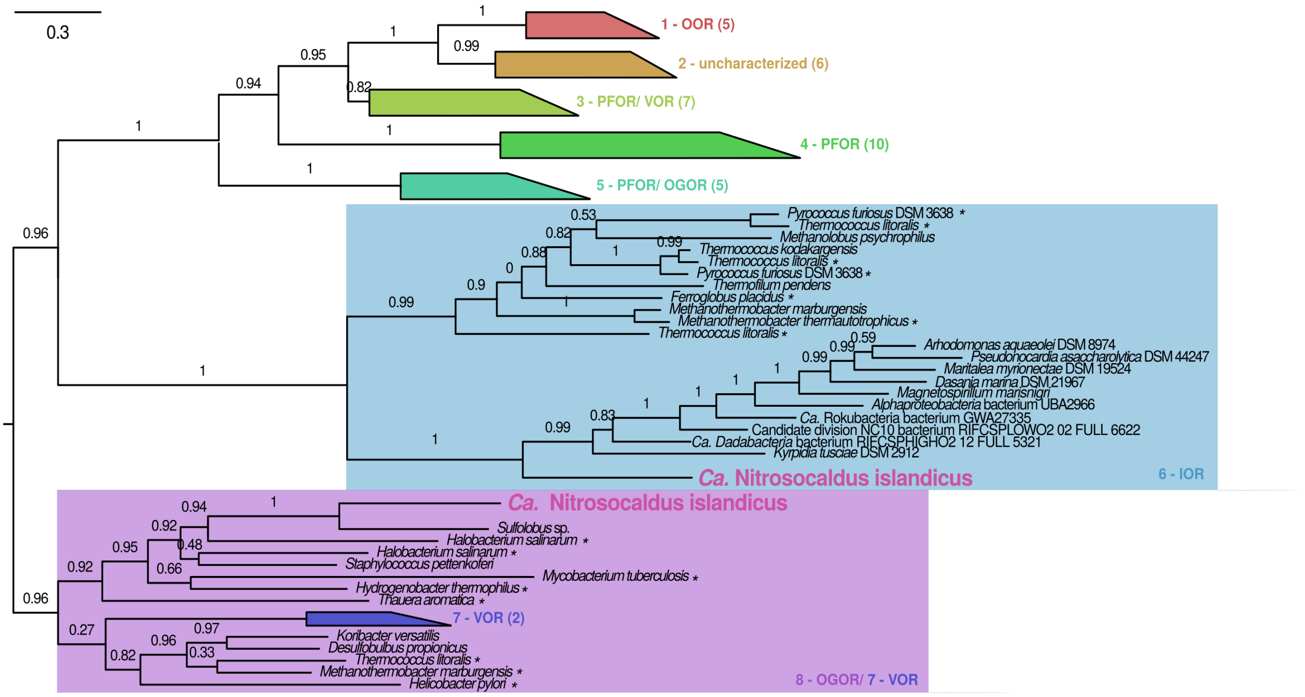

Fig. S5. Unrooted phylogenetic tree of 2-oxoacid oxidoreductases (OFORs) showing placement of the two OFORs present in "*Ca.* Nitrosocaldus islandicus strain 3F". Clade labels and most sequences are from (*30*). Numbers in brackets show the number of sequences in collapsed clades. Functionally characterized enzymes are indicated with a "*". OOR, oxalate oxidoreductase; PFOR, pyruvate:ferredoxin oxidoreductase; VOR, 2-ketoisovalerate oxidoreductase; OGOR, 2-oxoglutarate oxidoreductase; IOR, indolepyruvate oxidoreductase.

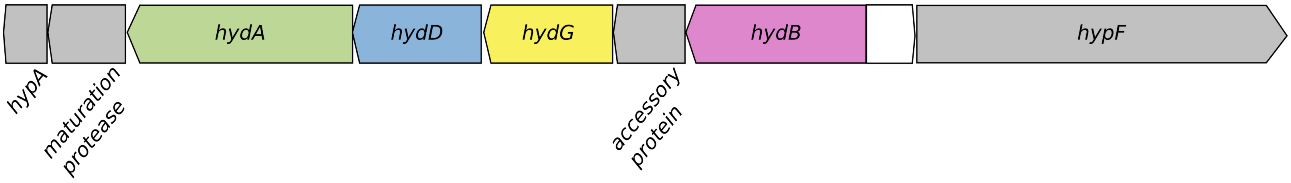

Figure S6. Schematic illustration of the genomic locus in "*Ca.* Nitrosocaldus islandicus" that encodes a bidirectional, NADP(H)-coupled type 3b [NiFe] hydrogenase. The locus contains the genes of the hydrogenase subunits hydADGB and of accessory proteins involved in enzyme maturation. Genes are drawn to scale. Locus tags (as found on MaGe) from left to right are as follows: AOA3F1_v2_1388, AOA3F1_v2_1387, AOA3F1_v2_1386, AOA3F1_v2_1385, AOA3F1_v2_1384, AOA3F1_v2_1383, AOA3F1_v2_1382, AOA3F1_v2_1381, AOA3F1_v2_1380.

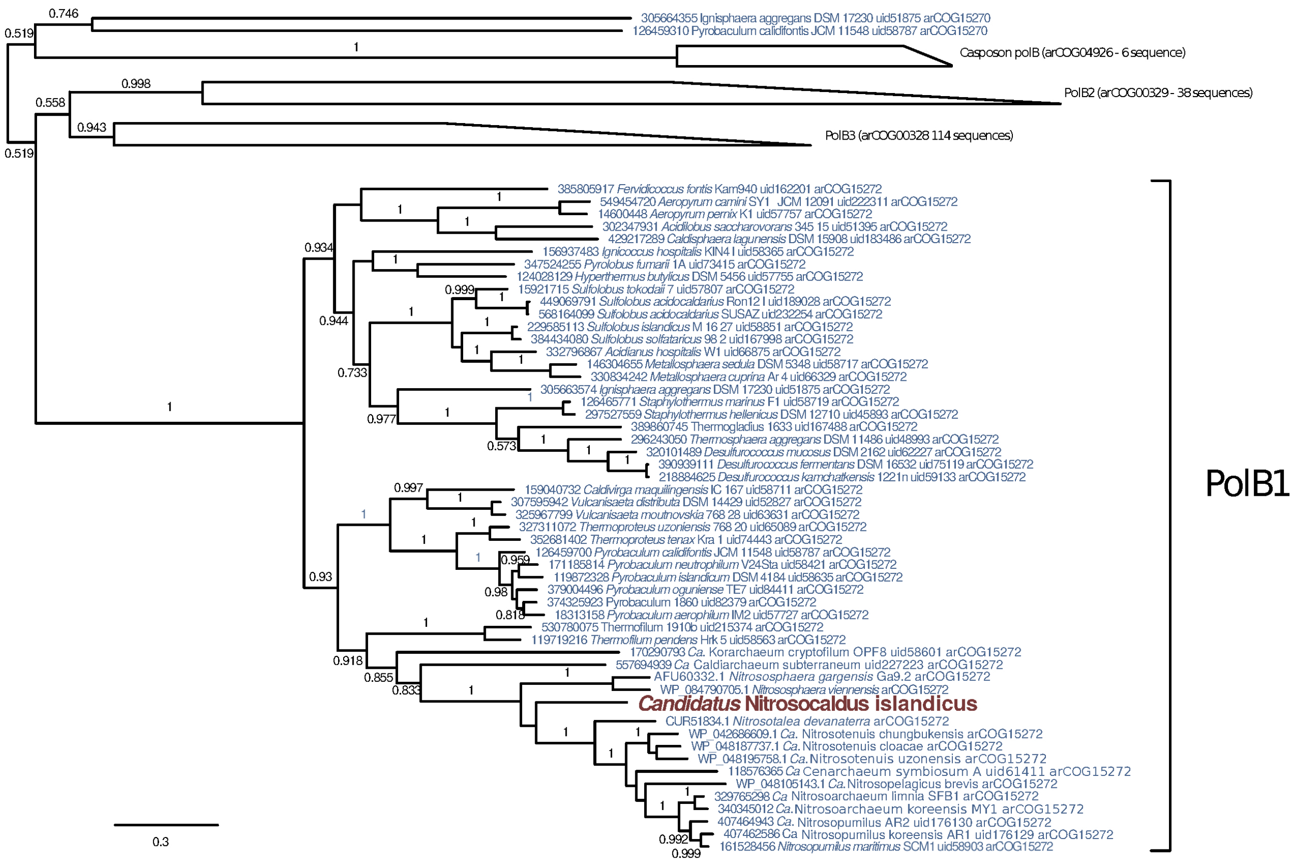

Fig S7. Unrooted approximate maximum likelihood tree showing placement of the PolB from "*Ca.* Nitrosocaldus islandicus" as a member of the PolB1 clade. The tree was calculated using FastTree[31] on 213 sequences aligned with mafft[32] (3058 aligned positions). Branch support greater than 0.5 is indicated on internal branches. PolB2, PolB3 and Casposon-related PolB (named according to *33*) have been collapsed into right trapezoids in which the bases indicate shortest and longest terminal branches within each clade.

References

1. Hallam, S. J. *et al.* Genomic analysis of the uncultivated marine crenarchaeote *Cenarchaeum symbiosum*. *Proc. Natl. Acad. Sci.* **103,** 18296–18301 (2006).
2. Lehtovirta-Morley, L. E. *et al.* Identifying potential mechanisms enabling acidophily in the ammonia-oxidising archaeon '*Candidatus* Nitrosotalea devanaterra'. *Appl. Environ. Microbiol.* AEM.04031-15 (2016). doi:10.1128/AEM.04031-15
3. Blainey, P. C., Mosier, A. C., Potanina, A., Francis, C. A. & Quake, S. R. Genome of a low-salinity ammonia-oxidizing archaeon determined by single-cell and metagenomic analysis. *PLoS ONE* **6,** (2011).
4. Zhalnina, K. V. *et al.* Genome sequence of *Candidatus* Nitrososphaera evergladensis from group I.1b enriched from Everglades soil reveals novel genomic features of the ammonia-oxidizing archaea. *PLoS ONE* **9,** e101648 (2014).
5. Butterfield, C. N. *et al.* Proteogenomic analyses indicate bacterial methylotrophy and archaeal heterotrophy are prevalent below the grass root zone. *PeerJ* **4,** e2687 (2016).
6. Kamanda Ngugi, D. *et al.* Comparative genomics reveals adaptations of a halotolerant *thaumarchaeon* in the interfaces of brine pools in the Red Sea. *ISME J.* **9,** 396–411 (2015).
7. Haroon, M. F., Thompson, L. R., Parks, D. H., Hugenholtz, P. & Stingl, U. A catalogue of 136 microbial draft genomes from Red Sea metagenomes. *Sci. Data* **3,** (2016).
8. Walker, C. B. *et al. Nitrosopumilus maritimus* genome reveals unique mechanisms for nitrification and autotrophy in globally distributed marine crenarchaea. *Proc. Natl. Acad. Sci. U. S. A.* **107,** 8818–8823 (2010).
9. Santoro, A. E. *et al.* Genomic and proteomic characterization of "*Candidatus* Nitrosopelagicus brevis": An ammonia-oxidizing archaeon from the open ocean. *Proc. Natl. Acad. Sci.* **112,** 1173–1178 (2015).
10. Kerou, M. *et al.* Proteomics and comparative genomics of *Nitrososphaera viennensis* reveal the core genome and adaptations of archaeal ammonia oxidizers. *Proc. Natl. Acad. Sci.* **113,** E7937–E7946 (2016).
11. Hug, L. A. *et al.* Critical biogeochemical functions in the subsurface are associated with bacteria from new phyla and little studied lineages. *Environ. Microbiol.* **18,** 159–173 (2016).

12. Ahlgren, N. A. *et al.* Genome and epigenome of a novel marine *Thaumarchaeota* strain suggest viral infection, phosphorothioation DNA modification and multiple restriction systems. *Environ. Microbiol.* n/a-n/a (2017). doi:10.1111/1462-2920.13768
13. Jung, M.-Y. *et al.* A hydrophobic ammonia-oxidizing archaeon of the *Nitrosocosmicus* clade isolated from coal tar-contaminated sediment. *Environ. Microbiol. Rep.* **8,** 983–992 (2016).
14. Mosier, A. C., Allen, E. E., Kim, M., Ferriera, S. & Francis, C. A. Genome sequence of "*Candidatus* Nitrosoarchaeum limnia" BG20, a low-salinity ammonia-oxidizing archaeon from the San Francisco Bay estuary. *J Bacteriol* **194,** 2119–2120 (2012).
15. Mosier, A. C., Allen, E. E., Kim, M., Ferriera, S. & Francis, C. A. Genome sequence of "*Candidatus* Nitrosopumilus salaria" BD31, an ammonia-oxidizing archaeon from the San Francisco Bay estuary. *J. Bacteriol.* **194,** 2121–2122 (2012).
16. Park, S.-J. *et al.* Draft genome sequence of an ammonia-oxidizing archaeon, "*Candidatus* Nitrosopumilus koreensis" AR1, from marine sediment. *J. Bacteriol.* **194,** 6940–6941 (2012).
17. Park, S. J. *et al.* Draft genome sequence of an ammonia-oxidizing archaeon, '*Candidatus* Nitrosopumilus sediminis' AR2, from Svalbard in the arctic circle. *J. Bacteriol.* **194,** 6948–6949 (2012).
18. Kim, B. K. *et al.* Genome sequence of an ammonia-oxidizing soil archaeon, "*Candidatus* Nitrosoarchaeum koreensis" MY1. *J. Bacteriol.* **193,** 5539–5540 (2011).
19. Bayer, B. *et al.* Physiological and genomic characterization of two novel marine *thaumarchaeal* strains indicates niche differentiation. *ISME J.* **10,** 1051–1063 (2016).
20. Sauder, L. A. *et al.* Cultivation and characterization of *Candidatus* Nitrosocosmicus exaquare, an ammonia-oxidizing archaeon from a municipal wastewater treatment system. *ISME J.* **11,** 1142–1157 (2017).
21. Li, Y. *et al.* A novel ammonia-oxidizing archaeon from wastewater treatment plant: Its enrichment, physiological and genomic characteristics. *Sci. Rep.* **6,** (2016).
22. Jung, M.-Y. *et al.* A mesophilic, autotrophic, ammonia-oxidizing archaeon of *thaumarchaeal* group I.1a cultivated from a deep oligotrophic soil horizon. *Appl. Environ. Microbiol.* **80,** 3645–3655 (2014).

23. Spang, A. *et al.* The genome of the ammonia-oxidizing *Candidatus* Nitrososphaera gargensis: insights into metabolic versatility and environmental adaptations. *Environ. Microbiol.* **14,** 3122–3145 (2012).

24. Hugerth, L. W. *et al.* Metagenome-assembled genomes uncover a global brackish microbiome. *Genome Biol.* **16,** 279 (2015).

25. Lebedeva, E. V. *et al.* Enrichment and genome sequence of the group I. 1a ammonia-oxidizing archaeon '*Ca.* Nitrosotenuis uzonensis' representing a clade globally distributed in thermal habitats. *Plos One* **8,** (2013).

26. Mende, D. R., Aylward, F. O., Eppley, J. M., Nielsen, T. N. & DeLong, E. F. Improved environmental genomes via integration of metagenomic and single-cell assemblies. *Front. Microbiol.* **7,** (2016).

27. Herbold CW, Lehtovirta-Morley LE, Jung M-Y, Jehmlich N, Hausmann B, Han P, *et al.* Ammonia-oxidising archaea living at low pH: Insights from comparative genomics. *Environ Microbiol*, in press (2017).

28. Stieglmeier M, Alves RJE, Schleper C. The Phylum Thaumarchaeota. In: Rosenberg E, DeLong EF, Lory S, Stackebrandt E, Thompson F (eds). *The Prokaryotes*. Springer Berlin Heidelberg, pp 347–362 (2014).

29. Pace HC and Brenner C. (2001). The nitrilase superfamily: classification, structure and function. *Genome Biol* **2**: reviews0001.1-reviews0001.9.

30. Gibson M, Chen P-T, Drennan CL. (2016). A structural phylogeny for understanding 2-oxoacid oxidoreductase function - ScienceDirect. *Curr Opin Struct Biol* **41**: 54–61.

31. Price, M. N., Dehal, P. S. & Arkin, A. P. FastTree: computing large minimum evolution trees with profiles instead of a distance matrix. *Mol. Biol. Evol.* **26,** 1641–50 (2009).

32. Katoh, K., Misawa, K., Kuma, K. & Miyata, T. MAFFT: a novel method for rapid multiple sequence alignment based on fast Fourier transform. *Nucleic Acids Res.* **30,** 3059–3066 (2002).

33. Makarova, K. S., Krupovic, M. & Koonin, E. V. Evolution of replicative DNA polymerases in archaea and their contributions to the eukaryotic replication machinery. *Front. Microbiol.* **5,** (2014).
